# Huntingtin Aggregate‐Responsive Autophagy Gene Circuit Mitigates Disease Pathology in R6/2 Mice

**DOI:** 10.1002/advs.78018

**Published:** 2026-09-27

**Authors:** Jie Zhu, Xi‐xiu Xie, Lei Li, Chen Tian, Hao‐tian Wang, Xiao‐jie Wang, Xiao‐lin Yu, Gui‐feng Zhang, Rui‐tian Liu

**Affiliations:** ^1^ National Key Laboratory of Biopharmaceutical Preparation and Delivery Institute of Process Engineering Chinese Academy of Sciences Beijing China; ^2^ University of Chinese Academy of Sciences Beijing China; ^3^ Department of Obstetrics and Gynecology National Clinical Research Center for Obstetric & Gynecologic Diseases Peking Union Medical College Hospital Chinese Academy of Medical Sciences and Peking Union Medical College Beijing China

**Keywords:** autophagy, Huntington's disease, immunoliposome, neuroprotection, polyglutamine, synthetic gene circuit, TFEB

## Abstract

The accumulation of mutant huntingtin (mHTT) aggregates drives the pathology of Huntington's disease (HD), yet therapies capable of distinguishing toxic species from wild‐type proteins remain elusive. Here, a synthetic gene circuit, termed ARAA, was engineered to couple the preferential recognition of aggregated polyQ species to the on‐demand activation of autophagy. Utilizing a repurposed bacterial NarX–NarL system fused with a conformation‐sensitive intrabody, the circuit detects pathological polyQ conformers and triggers the transcriptional expression of the master autophagy regulator TFEB. To enable systemic application, the ARAA plasmid is encapsulated in CD98‐targeted immunoliposomes (LIP‐CD98) that facilitate efficient blood‐brain barrier crossing via receptor‐mediated transcytosis. In the R6/2 HD mouse model, ARAA treatment significantly reduces mHTT burden, attenuates neuroinflammation, and rescues synaptic deficits. This closed‐loop intervention improves motor function and extends lifespan. Together, these findings provide proof‐of‐concept evidence that aggregate‐responsive regulation of autophagy can mitigate disease‐associated phenotypes in exon 1‐based HD models.

## Graphical abstract

CD98‐mediated receptor‐mediated transcytosis enables LIP‐CD98 nanocarriers to cross the blood–brain barrier and deliver ARAA to neurons. mHTT aggregates activate the 11G–NarX sensor, initiating Auto‐P and Trans‐P signaling through the VP48–NarL relay. This response conditionally induces TFEB expression and promotes aggregate clearance, supporting a proof‐of‐concept closed‐loop gene‐regulation strategy for Huntington's disease.

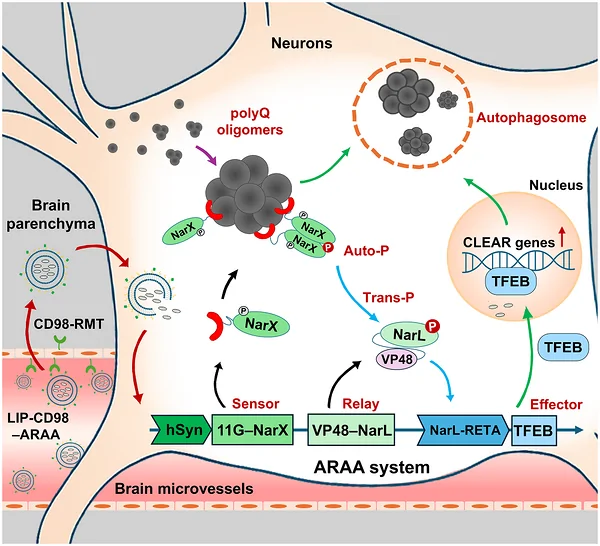

## 1 Introduction

Huntington's disease (HD) is an autosomal‐dominant neurodegenerative disorder caused by a CAG expansion in the *HTT* gene, producing mutant huntingtin (mHTT). Proteolytic cleavage of mHTT generates toxic N‐terminal fragments (e.g., exon 1) containing the elongated polyglutamine (polyQ) tract, which rapidly misfold, aggregate, and drive selective neurodegeneration in the striatum and cortex [1, 2]. mHTT aggregation is a central pathological hallmark of HD and initiates widespread cellular dysfunction, including synaptic pathology, mitochondrial impairment, neuroinflammation, and ultimately neuronal loss. A major determinant of this toxicity is impaired proteostasis, particularly dysfunction of the autophagy–lysosomal pathway (ALP), a core degradative system responsible for eliminating misfolded and aggregated proteins [2, 3]. Accumulating mHTT disrupts the autophagy–lysosomal pathway at multiple steps, including cargo recognition, autophagosome trafficking, and maturation, thereby compromising degradative efficiency. Conversely, impaired autophagy promotes further mHTT accumulation and aggregation, establishing a self‐reinforcing pathogenic cycle [4, 5, 6, 7]. The reciprocal relationship between mHTT aggregation and ALP failure has emerged as a mechanistic nexus driving HD progression. Therefore, therapeutic strategies that concurrently target mHTT aggregates and augment autophagy hold promise for the treatment of HD.

Current efforts to reduce mHTT burden have focused substantially on gene‐silencing strategies, including antisense oligonucleotides (ASOs) and RNA interference (RNAi), which have demonstrated robust suppression of HTT expression and therapeutic benefits in preclinical HD models [8, 9]. Despite these advances, clinical translation has been challenging. The Phase III trial of the ASO tominersen was terminated due to futility and a concerning trend toward worse motor and functional outcomes in high‐dose cohorts [2]. These dose‐dependent adverse effects highlight a key limitation of non‐selective HTT suppression, which indiscriminately reduces both mutant and wild‐type HTT. Because wild‐type HTT supports neuronal survival, synaptic vesicle trafficking, and synaptic connectivity, its unintended depletion could limit the therapeutic benefits of non‐allele‐selective HTT‐lowering strategies [10, 11]. Moreover, gene‐silencing approaches primarily prevent new protein synthesis and do not eliminate pre‐existing mHTT aggregates, which accumulate over decades and remain a major source of cytotoxicity in symptomatic patients. These limitations underscore the need for strategies that selectively reduce mHTT aggregates.

Transcription factor EB (TFEB) is a master transcriptional regulator of the autophagy–lysosomal system. By coordinately driving the expression of lysosomal and autophagy genes such as *p62* and *LC3B*, TFEB enhances autophagic flux and degradative capacity, thereby maintaining cellular homeostasis and playing a critical role in clearing pathological protein aggregates and mitigating neuronal stress [12]. Previous reports have shown that TFEB levels are decreased in HD patients. Existing TFEB‐targeting strategies, including small molecules and AAV‐mediated overexpression, stimulate autophagy and lysosome activity, and lower mHTT in R6/2 HD mice. However, TFEB‐centered therapeutic strategies in HD lack spatial and temporal control, and persistent or excessive TFEB activation may impose context‐dependent cellular stress or toxicity [13, 14].

To achieve the precise regulation of TFEB, we here apply a bacterial NarX–NarL two‐component system, which features an orthogonal architecture that can be engineered to convert specific molecular cues into tailored transcriptional programs. Its intrinsic signal amplification and switch‐like dynamics enable sensitive activation with minimal background, making it particularly well‐suited for implementing conditional and self‐limiting TFEB activation in response to mHTT aggregates. Building upon this rationale, we developed an “Aggregate‐Responsive Autophagy Activator” (ARAA) gene circuit. In this system, we re‐engineered NarX to function as a sensor for mHTT oligomers and re‐purposed NarL to drive TFEB expression. This closed‐loop design was intended to upregulate autophagy in response to aggregate accumulation and to reduce effector activity as the aggregate burden declined, thereby limiting sustained TFEB overexpression. We also evaluated the therapeutic efficacy and safety of this system in a mouse model of HD.

## 2 Results and Discussion

### 2.1 Aggregate‐Responsive Sensing Enables Conditional TFEB Expression and Reduces mHTT Exon 1 Burden

**FIGURE 1:**
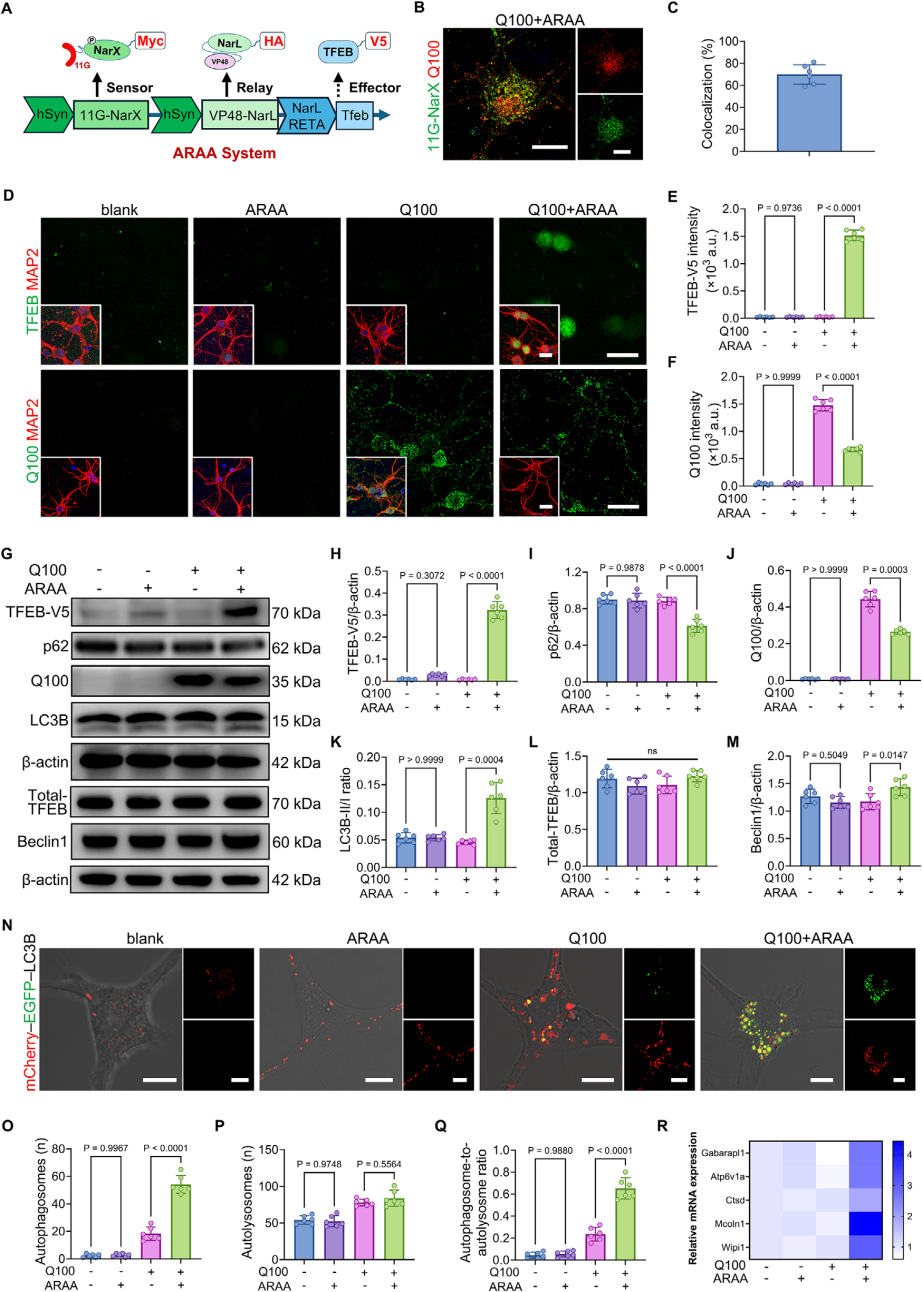
The ARAA system senses Q100 aggregates to trigger TFEB expression and autophagic clearance in neurons. Primary cortical neurons were transfected with the Aggregate‐Responsive Autophagy Activator (ARAA) plasmids, negative control (NC; empty pcDNA3.1), and/or Httex1‐Q100 (Q100) at day in vitro 7 (DIV7) and analyzed at DIV10. The Q100 plasmid encodes a pathogenic HTT exon 1 fragment comprising the N17 domain, an expanded polyglutamine tract containing 100 repeats, and the proline‐rich domain, fused to a C‐terminal 6×His tag for immunodetection. (A) Schematic illustration of the ARAA plasmid constructs. The sensor (11G–NarX) and relay (VP48–NarL) modules are independently regulated by the hSyn promoter and carry C‐terminal Myc and HA tags, respectively. The effector module, TFEB, is conditionally driven by a synthetic NarL‐responsive promoter (NarL‐RETA) and carries a V5 tag. (B) Representative immunofluorescence images showing the colocalization of the 11G–NarX ARAA sensor (green) with Q100 aggregates (red) in co‐transfected primary neurons. Scale bar, 10 µm. (C) Quantification of the colocalization between 11G–NarX and Q100 based on the images in (B), calculated as the merged area relative to the 11G–NarX‐positive area. (D) Representative immunofluorescence images showing the expression of the ARAA effector TFEB, detected using the V5 tag, and Q100, detected using the His tag, in primary neurons from the indicated treatment groups. Scale bar, 20 µm. (E, F) Quantification of TFEB (E) and Q100 (F) fluorescence intensities based on the images in (D) using ImageJ software. (G) Representative Western blots showing the protein levels of ARAA‐derived TFEB, detected using the V5 tag, total TFEB, Q100, and the autophagy‐related proteins LC3B, p62, and Beclin 1 in primary neurons; β‐actin served as a loading control. (H–M) Quantification of relative protein levels of V5‐tagged TFEB (H), p62 (I), Q100 (J), the LC3‐II/LC3‐I ratio (K), total TFEB (L), and Beclin 1 (M) based on the blots in (G). (N) Representative live‐cell fluorescence images of primary neurons expressing the tandem mCherry–EGFP–LC3B reporter in the indicated treatment groups. Yellow puncta represent autophagosomes, whereas red‐only puncta represent autolysosomes. Scale bar, 10 µm. (O–Q) ImageJ‐based quantification of the number of yellow autophagosomes (O), red‐only autolysosomes (P), and the autophagosome‐to‐autolysosome ratio (Q) per cell based on the images in (N). (R) RT‐qPCR analysis of representative MiT/TFE target genes, including *Wipi1, Mcoln1, Ctsd, Atp6v1a*, and *Gabarapl1*, in primary neurons (*n* = 3 biologically independent replicates). All data are presented as mean ± SD; *n* = 6 biologically independent replicates unless otherwise noted. Statistical analysis was performed using one‐way ANOVA, and specific comparisons between groups of interest are indicated. Exact *p* values are shown in the figure unless *p* < 0.0001.

To enable aggregate‐responsive activation of autophagy pathways, we engineered a three‐module ARAA circuit designed to conditionally drive TFEB expression in the presence of pathogenic polyQ‐expanded HTT exon 1 aggregates (Figure 1A). The system, constructed on a pcDNA3.1 backbone, comprises a sensor, a relay, and an effector module. The sensor consists of the aggregate‐recognizing 11G scFv fused to a truncated NarX histidine kinase. In this configuration, binding to polyQ‐containing aggregates is designed to induce a conformational change that activates the NarX signaling domain. This activation initiates a phosphotransfer cascade to the relay module, which comprises the cognate response regulator NarL fused to VP48, a potent synthetic transactivation domain. Upon phosphorylation, the VP48–NarL chimera dimerizes and binds to the effector module, a synthetic promoter harboring tandem NarL‐binding sites upstream of a minimal TA promoter, thereby initiating the transcription of C‐terminal V5‐tagged mouse TFEB. To confer neuronal specificity, expression of both the 11G–NarX sensor and the VP48–NarL relay is driven by the human synapsin (hSyn) promoter, whereas TFEB expression is strictly gated by the NarL‐responsive synthetic promoter. Optimized Kozak sequences were included upstream of all coding regions, and distinct epitope tags (Myc, HA, and V5) were appended to facilitate immunodetection of each component.

We first characterized the aggregate‐recognizing 11G scFv. To distinguish non‐pathogenic from pathogenic polyQ states, Q6 and Q23 served as predominantly non‐aggregating controls, whereas Q53 and Q100 represented pathogenic polyQ‐expanded species. Q53 was used for biochemical characterization because longer polyQ tracts are less tractable in prokaryotic expression systems [15], while Q100 provided a robust aggregation‐prone model for mammalian‐cell studies. The GST fusion maintained Httex1‐Q53 in a predominantly soluble state, whereas proteolytic removal of GST promoted rapid aggregation of the Q53 moiety. Dot‐blot analysis showed that 11G preferentially bound the cleaved, highly aggregated Httex1‐Q53 preparation relative to soluble GST and uncleaved GST‐Httex1‐Q53 controls (Figure S1A), supporting preferential recognition of aggregated Httex1‐Q53 under the tested conditions. The binding affinity of 11G for Httex1‐Q53 aggregates was further assessed by ELISA, yielding an EC50 of approximately 21.6 nm (Figure S1B). Consistently, dot‐blot analysis of total protein extracts from primary neurons showed robust 11G reactivity in samples expressing Httex1‐Q100, which contained a heterogeneous mixture of monomers, oligomers, and higher‐order aggregates (Figure S1C), whereas no detectable signal was observed in samples expressing the predominantly non‐aggregating Q6 or Q23 variants. These results support preferential recognition of expanded polyQ‐associated aggregated species in this cellular context. Furthermore, co‐immunoprecipitation using 11G as bait, followed by native PAGE, enriched high‐molecular‐weight polyQ assemblies spanning approximately 440–800 kDa from R6/2 HD mouse brain homogenates (Figure S1D), further supporting the recognition of oligomeric and aggregated polyQ species by 11G.

To translate binding into an intracellular trigger, 11G was fused to NarX to generate the ARAA sensor (11G–NarX). Colocalization of the sensor and relay modules with MAP2 indicated an ARAA transfection efficiency of approximately 60% in primary neurons (Figure S1E,F). Moreover, 11G–NarX co‐localized with Httex1‐Q100 aggregates in co‐transfected neurons, confirming in situ target engagement (Figure 1B,C). Next, we examined component expression and conditional effector activation. Immunofluorescence analysis showed that both the sensor (11G–NarX) and the relay (VP48–NarL) were robustly expressed in neurons; importantly, co‐expression with Q100 did not alter the expression levels of these components compared to ARAA alone (Figure S1G–L).

Under basal conditions, the ARAA sensor and relay were readily detectable, whereas circuit‐derived V5‐tagged TFEB remained at a low level, indicating a quiescent “off” state. By contrast, co‐expression with Httex1‐Q100 switched the circuit “on,” resulting in a marked increase in V5‐tagged TFEB expression. Immunofluorescence analysis confirmed the induction of ARAA‐derived TFEB and showed a concomitant reduction in Q100 levels in neurons co‐expressing Q100 and ARAA (Figure 1D–F). Consistent with these observations, Western blot analysis showed increased V5‐tagged TFEB and decreased p62 and Q100 levels in the Q100+ARAA group, together with an elevated LC3‐II/LC3‐I ratio (Figure 1G–K). In contrast, total TFEB protein levels were comparable among the treatment groups (Figure 1G,L), whereas Beclin 1 showed a modest increase in neurons co‐expressing Q100 and ARAA (Figure 1G,M).

We next examined autophagosome and autolysosome dynamics using the tandem mCherry–EGFP–LC3B reporter. Neurons co‐expressing Q100 and ARAA exhibited a significant increase in yellow puncta, indicating an increased abundance of autophagosomes. Red‐only autolysosomal puncta and the autophagosome‐to‐autolysosome ratio were quantified in parallel (Figure 1N–Q). To further evaluate autophagic flux, primary neurons were treated with bafilomycin A1 (BafA1) to inhibit lysosomal acidification and autophagosome degradation. In neurons co‐expressing Q100 and ARAA, BafA1 treatment induced further accumulation of LC3‐II and increased p62 levels relative to the corresponding untreated group (Figure S1M–P). These findings support the interpretation that the reduced p62 level and increased LC3‐II/LC3‐I ratio observed following ARAA activation are associated with enhanced autophagic flux rather than a simple blockade of downstream autophagic degradation. Reverse transcription quantitative PCR (RT‐qPCR) analysis showed increased expression of representative MiT/TFE target genes within the CLEAR network, including *Wipi1, Mcoln1, Ctsd, Atp6v1a*, and *Gabarapl1*, in neurons co‐expressing Q100 and ARAA (Figure 1R). Collectively, these results support aggregate‐dependent activation of ARAA‐derived TFEB and indicate that this response is associated with enhanced autophagic processing and reduced Q100 levels, whereas neurons lacking Q100 retain the circuit in a largely quiescent state.

Finally, the reversibility of ARAA‐derived TFEB expression was evaluated using a Tet‐Off Httex1‐Q100 system in primary neurons co‐transduced with LV‐Httex1‐Q100 and LV‐ARAA. Neurons were initially cultured in the absence of doxycycline to permit Q100 expression and activation of the ARAA circuit. Following the addition of doxycycline to suppress de novo Q100 synthesis, immunofluorescence and Western blot analyses showed that V5‐tagged TFEB progressively declined and returned toward baseline over the subsequent seven‐day period (Figure S2A–E). In contrast, TFEB expression remained sustained in cultures maintained without doxycycline.

To determine whether the reduction in ARAA‐derived TFEB was accompanied by removal of the polyQ trigger, dot blot analysis was subsequently performed using the 1C2 antibody. In the absence of doxycycline, 1C2‐reactive expanded polyQ‐containing Q100 species increased progressively throughout the seven‐day observation period. By contrast, following doxycycline treatment, the 1C2‐reactive signal was markedly reduced by day 3 and became undetectable at days 5 and 7 (Figure S2F,G). These findings demonstrate that suppression and subsequent depletion of the Q100 trigger are accompanied by the return of ARAA‐derived TFEB expression to a dormant state, supporting the reversible and closed‐loop behavior of the circuit.

### 2.2 ARAA Mitigates PolyQ‐Induced Synaptic Damage and Neuronal Death In Vitro

**FIGURE 2:**
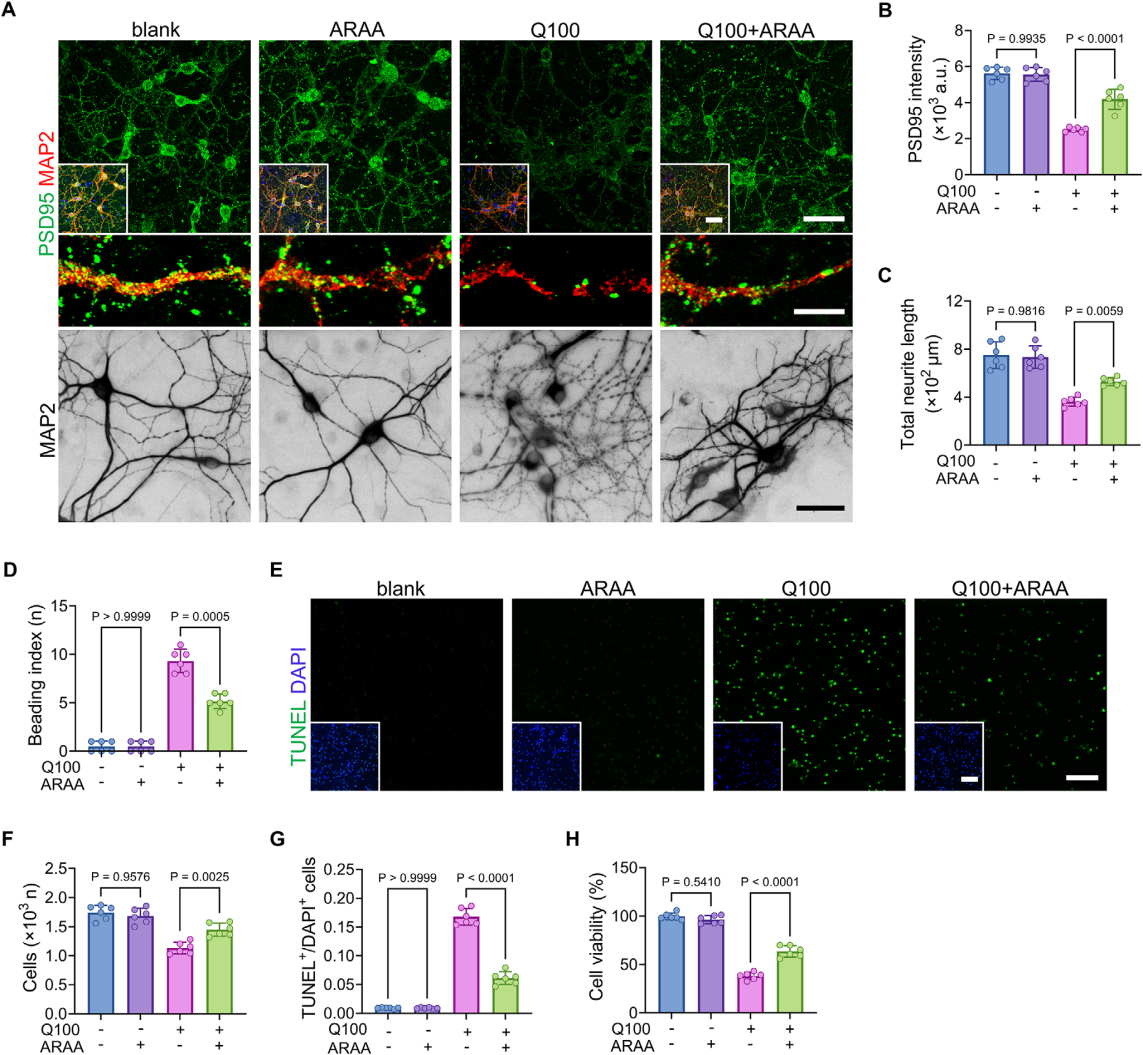
ARAA treatment mitigates Q100‐induced synaptic damage and neurotoxicity in primary cortical neurons. (A) Representative immunofluorescence images showing PSD95 expression co‐stained with MAP2, together with separate MAP2 staining to assess neuronal dendritic morphology, in primary neurons from the indicated treatment groups. For PSD95/MAP2 co‐staining, scale bars are 30 µm for overview images and 10 µm for magnified views; for separate MAP2 staining, the scale bar is 30 µm. (B) Quantification of PSD95 fluorescence intensity based on the PSD95/MAP2 co‐staining images in (A) using ImageJ software. (C, D) Quantification of total neurite length per cell (C) and the number of neuritic varicosities (bead‐like swellings) (D) based on (A) using ImageJ software. (E) Representative immunofluorescence images of TUNEL staining (apoptosis) and DAPI (nuclear) staining. Scale bar, 100 µm. (F, G) Quantification of total DAPI‐positive cell counts (F) and the percentage of TUNEL‐positive cells (G) from (E) using ImageJ software. (H) Neuronal viability assessed by MTT assay. All data are presented as mean ± SD; *n* = 6 biologically independent replicates. Statistical analysis was performed using one‐way ANOVA, and specific comparisons between groups of interest are indicated. Exact *p* values are shown in the figure unless *p* < 0.0001.

We next examined whether ARAA‐mediated autophagy activation confers neuroprotection against mHTT toxicity. Immunostaining revealed that Q100 expression significantly reduced PSD95 signal intensity and puncta density compared with controls, whereas ARAA co‐expression preserved PSD95‐positive synaptic clusters and yielded higher PSD95 intensity per neuron relative to the Q100‐only group (Figure 2A,B). Moreover, MAP2‐positive dendrites of the neurons with Q100 expression appeared dystrophic and beaded (exhibiting varicosities), while neurites in ARAA‐treated neurons were smoother and more continuous, with markedly fewer swellings (Figure 2A,C). Quantitative morphometric analysis confirmed that ARAA restored total neurite length per cell and reduced the density of beaded varicosities compared with Q100‐only neurons (Figure 2A,D), demonstrating the protection of ARAA against mHTT‐induced neuritic degeneration.

We further investigated the effects of ARAA on neuronal survival under polyQ stress. TUNEL assays revealed substantial apoptosis in neurons expressing Q100, whereas ARAA co‐expression markedly lowered the percentage of apoptotic cells (Figure 2E–G). Consistently, MTT assays showed that Q100 diminished neuronal viability relative to controls, whereas ARAA significantly rescued this viability deficit (Figure 2H). Collectively, these findings demonstrate that ARAA counteracts mHTT‐induced synaptotoxicity and cell death in vitro, likely by enhancing the autophagic clearance of toxic protein species, thereby preserving neuronal structure and function.

### 2.3 Efficient Delivery of ARAA Gene to Neurons by Brain‐Targeted Liposomes

**FIGURE 3:**
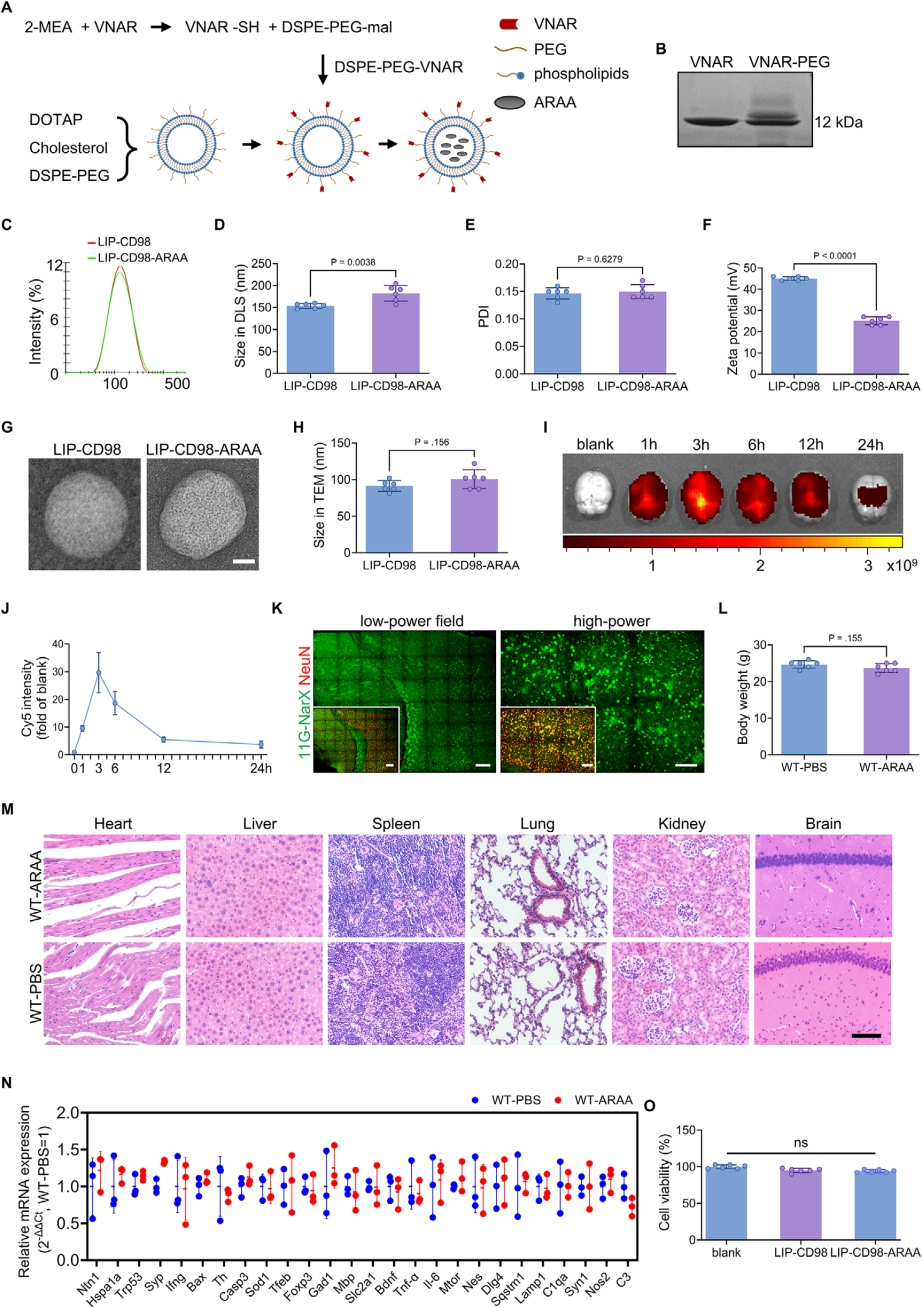
Construction, characterization, and safety evaluation of the brain‐targeting LIP‐CD98‐ARAA nanocarrier. (A) Schematic illustration of cationic liposome preparation, antibody conjugation, and ARAA plasmid encapsulation to form LIP‐CD98‐ARAA. (B) Coomassie Brilliant Blue staining to assess the PEGylation efficiency of the anti‐CD98 nanobody (*n* = 3). (C–F) Dynamic light scattering (DLS) characterization: representative size distribution profiles of LIP‐CD98 before and after plasmid loading (C), and quantification of hydrodynamic diameter (D), polydispersity index (PDI) (E), and Zeta potential of LIP‐CD98‐ARAA (F). (G, H) Representative transmission electron microscopy (TEM) images of brain‐targeted liposomes before (Empty LIP‐CD98) and after ARAA plasmid loading (LIP‐CD98‐ARAA) (G); scale bar, 20 nm. The corresponding particle size distribution was derived from TEM analysis using ImageJ software (H). (I) In vivo whole‐brain fluorescence imaging at 0, 1, 3, 6, 12, and 24 h post‐injection of Cy5‐labeled LIP‐CD98 encapsulating the ARAA plasmid (Ex/Em: 640/680 nm). (J) Quantification of brain fluorescence intensity from (I), normalized to the blank control (*n* = 3). (K) Immunofluorescence images of the mouse cortex and striatum 48 h post‐injection, showing the expression of the Myc‐tagged ARAA sensor (green) within NeuN‐positive neurons (red). Scale bars: 200 µm (overview) and 100 µm (magnified view) (*n* = 3). (L–N) In vivo safety assessment following administration of a high‐dose regimen of LIP‐CD98‐ARAA (three times the therapeutic dose, injected every three days for one month). (L) Body weight statistics measured after 30 days of administration. (M) Representative H&E staining images of major organs (scale bar, 200 µm). (N) RT‐qPCR analysis of genes related to neurodevelopment, inflammation, and autophagy in whole‐brain tissue (*n* = 3). (O) MTT assay evaluating neurotoxicity in SH‐SY5Y cells treated with 50 µg/mL LIP‐CD98‐ARAA. Data are presented as mean ± SD; *n* = 6 biologically independent replicates unless otherwise noted. Statistical analysis was performed using unpaired two‐tailed t‐tests for (D–F, H, L) and one‐way ANOVA for (O). Exact *p* values are shown in the figure, except where *p* < 0.0001.

A major barrier to CNS gene therapy is efficient delivery of genes across the blood‐brain barrier (BBB). CD98 heavy chain (CD98hc) is a key transport receptor that facilitates efficient transcytosis across the BBB [16]. To leverage this pathway for brain‐specific delivery, we conjugated an anti‐CD98 single‐domain antibody (VNAR) to the surface of liposomes encapsulating the ARAA plasmids, designated as LIP‐CD98‐ARAA. As shown in Figure 3A, cationic liposomes were PEGylated with a maleimide–PEG–linked VNAR against CD98hc and loaded with ARAA DNA. SDS–PAGE confirmed efficient VNAR conjugation and incorporation into PEGylated liposomes (Figure 3B). Dynamic light scattering (DLS) showed that the hydrodynamic diameters of empty LIP‐CD98 and plasmid‐loaded LIP‐CD98‐ARAA ranged from 150 to 170 nm (Figure 3C,D), exhibiting a narrow particle size distribution with a polydispersity index (PDI) of approximately 0.15 (Figure 3E). The zeta potential decreased from approximately +45 mV (empty LIP‐CD98) to +25 mV upon DNA loading (Figure 3F). This remaining net positive charge is consistent with preserved colloidal stability and cellular uptake competence. Transmission electron microscopy (TEM) results demonstrated that both empty LIP‐CD98 and LIP‐CD98‐ARAA formed uniform nanoparticles with diameters of approximately 100 nm (Figure 3G,H). Together, these data indicate that the CD98hc‐targeted particles (LIP‐CD98) are well‐defined carriers for plasmid payloads.

We next evaluated brain delivery of LIP‐CD98‐ARAA in vivo. Following intravenous administration of Cy5‐labeled LIP‐CD98, whole‐brain fluorescence was robust relative to background controls within 24 h (Figure 3I,J). Given the limited brain penetration of many systemically administered macromolecular and nanoparticle formulations [17, 18], the increased brain‐associated fluorescence observed with LIP‐CD98 is consistent with enhanced brain delivery following CD98hc targeting. Importantly, immunostaining for the ARAA sensor (Myc‐tagged 11G–NarX) demonstrated expression in NeuN‐positive neurons in both the cortex and striatum (Figure 3K). Cell‐type profiling in the striatum further showed that 11G–NarX colocalized predominantly with neuronal markers, with approximately 85% overlap with NeuN and 46% with MAP2. Approximately 10% overlap was observed with GFAP, whereas colocalization with Iba1 or OLIG2 was below 2% (Figure S3A,B). The modest GFAP overlap may partly reflect the close spatial association of astrocytic processes with neuronal somata and neurites in two‐dimensional tissue sections, although limited expression in astrocytes cannot be excluded. Overall, these findings support preferential neuronal expression following systemic LIP‐CD98‐ARAA administration.

The biocompatibility of the delivery system was evaluated in wild‐type mice. To assess potential in vivo side effects, mice received a high‐dose regimen of LIP‐CD98‐ARAA (three times the therapeutic dose; injection volume < 300 µL per mouse) administered every three days for one month. No overt clinical signs of distress were observed in LIP‐CD98‐ARAA‐treated mice throughout the study, and body weight measured after the 30‐day administration period was comparable to that of controls (Figure 3L). H&E staining exhibited no appreciable histological abnormalities in major organs (heart, liver, spleen, lung, kidney, and brain) (Figure 3M). Furthermore, RT‐qPCR analysis of brain tissue showed no significant changes in the expression of genes related to neurodevelopment, inflammation, or autophagy (Figure 3N), suggesting that the treatment with LIP‐CD98‐ARAA did not produce detectable changes in the safety‐related endpoints assessed under the present experimental conditions on these pathways. Consistently, MTT assays revealed that LIP‐CD98‐ARAA even at 50 µg/mL did not show obvious cytotoxicity to SH‐SY5Y cells (Figure 3O).

### 2.4 Systemic ARAA Ameliorates Motor and Anxiety‐Like Phenotypes and Extends Survival in R6/2 HD Mice

**FIGURE 4:**
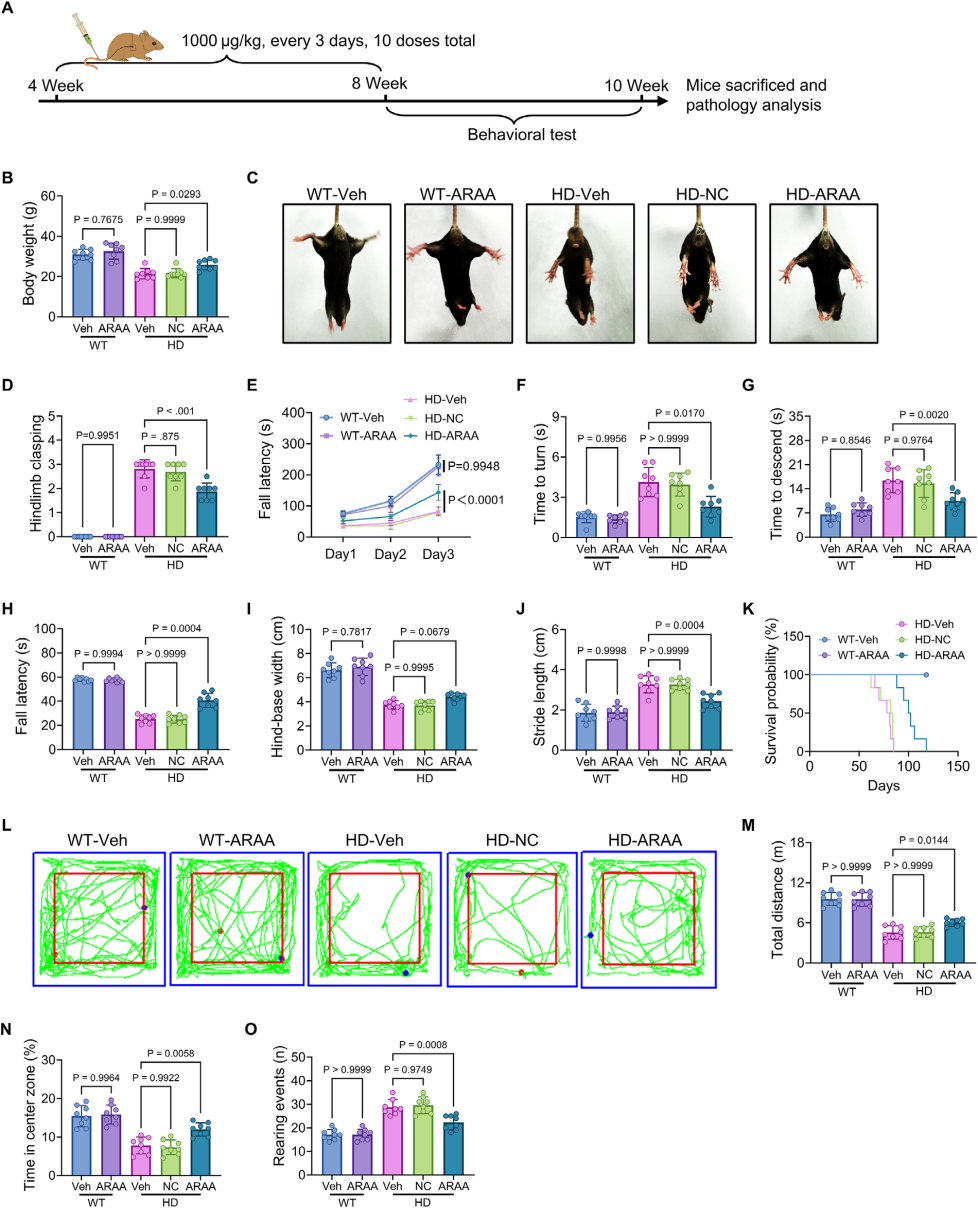
ARAA treatment improves motor function, improves open‐field exploratory and anxiety‐related measures, and extends lifespan in R6/2 HD mice. Four‐week‐old R6/2 transgenic HD mice (expressing exon 1 of HTT with ∼ 150 CAG repeats, Q150) and wild‐type (WT) littermates received intravenous injections of the indicated formulations every 3 days for 4 weeks. Mice were assigned to the following treatment groups: HD‐Veh (PBS), HD‐NC (LIP‐CD98 encapsulating empty pcDNA3.1), HD‐ARAA (LIP‐CD98‐ARAA), WT‐Veh (PBS), and WT‐ARAA (LIP‐CD98‐ARAA) (*n* = 8 per group). Behavioral tests were conducted at 8–10 weeks of age. (A) Experimental timeline of treatment and assessments. (B) Body weight measured at the end of the treatment period. (C, D) Representative photographs showing hindlimb clasping phenotypes (C) and the corresponding clinical clasping scores (D). (E) Latency to fall in the rotarod test. (F, G) Time to orient downward (F) and time to descend completely (G) in the pole test. (H) Latency to fall in the wire hang test. (I, J) Quantitative gait analysis of hind‐base width (I) and stride length (J), based on eight consecutive footprint intervals recorded along a 50‐cm walking track for each mouse. (K) Kaplan–Meier survival curves of R6/2 mice. (L) Representative 5‐min movement traces in the open‐field test. (M–O) Assessment of locomotor and anxiety‐related behaviors in the open‐field test: total distance traveled (M), percentage of the total test time spent in the center zone (N), and number of vertical rearing events (O). All quantitative data are presented as mean ± SD; *n* = 8 mice per group. Statistical significance was determined by one‐way ANOVA for (B, D, F–J, M–O), two‐way ANOVA for the rotarod test in (E), and the log‐rank (Mantel–Cox) test for the survival analysis in (K). Specific comparisons between groups of interest are indicated. Exact *p* values are shown in the figure unless *p* < 0.0001.

We next investigated whether ARAA could ameliorate motor deficits in R6/2 HD mice. Male R6/2 HD mice (4 weeks old) were randomly assigned to three groups: HD‐ARAA (injected with LIP‐CD98‐ARAA), HD‐NC (injected with LIP‐CD98‐pcDNA3.1 negative control), and HD‐Veh (injected with PBS). Wild‐type littermates injected with PBS (WT‐Veh) or LIP‐CD98‐ARAA (WT‐ARAA) served as controls (Figure 4A). All injections were administered intravenously once every 3 days for 4 weeks.

Compared with HD‐Veh and HD‐NC mice, HD‐ARAA mice maintained significantly higher body weight (Figure 4B). To assess motor performance, we conducted a battery of behavioral tests. HD‐Veh and HD‐NC mice displayed the characteristic clasping reflex, whereas HD‐ARAA mice frequently exhibited a normal splayed posture (Figure 4C), resulting in significantly lower clasping scores (Figure 4D). In the rotarod assay, HD‐ARAA mice remained on the accelerating rod for a significantly longer duration than HD controls (HD‐Veh and HD‐NC), indicating preserved motor coordination and endurance (Figure 4E). In the pole test, HD‐Veh and HD‐NC mice exhibited delayed descent and turning compared with WT‐Veh mice. HD‐ARAA treatment significantly reduced both movement initiation (T‐turn) and descending time (Figure 4F,G). Neuromuscular strength was similarly improved, as evidenced by a prolonged latency of the HD‐ARAA group to fall in the wire‐hang test (Figure 4H).

Moreover, quantitative gait analysis revealed a trend toward reduced hind‐base width in HD‐ARAA mice, although the difference did not reach statistical significance (Figure 4I). By contrast, stride length was significantly increased compared with the HD‐Veh and HD‐NC groups (Figure 4J). Kaplan–Meier analysis further showed that HD‐Veh and HD‐NC mice had similar mean survival times of 77 days, whereas ARAA treatment extended the mean survival time to 102 days (Figure 4K). Given that R6/2 HD mice typically exhibit reduced exploratory activity and anxiety‐like behavior, we next performed the open‐field test. Compared with WT‐Veh mice, HD‐Veh and HD‐NC mice traveled shorter distances, spent a lower percentage of the total test time in the center zone, and exhibited altered vertical rearing activity (Figure 4L–O). HD‐ARAA treatment increased the total distance traveled and the percentage of time spent in the center zone, while reducing the number of vertical rearing events (Figure 4M–O). Collectively, these behavioral and survival findings indicate that systemic delivery of ARAA by LIP‐CD98 improves several measures of motor and exploratory performance, extends survival in R6/2 HD mice, and attenuates anxiety‐like behavior.

### 2.5 ARAA Promotes Clearance of mHTT Aggregates via Activation of the TFEB‐Autophagy Pathway

**FIGURE 5:**
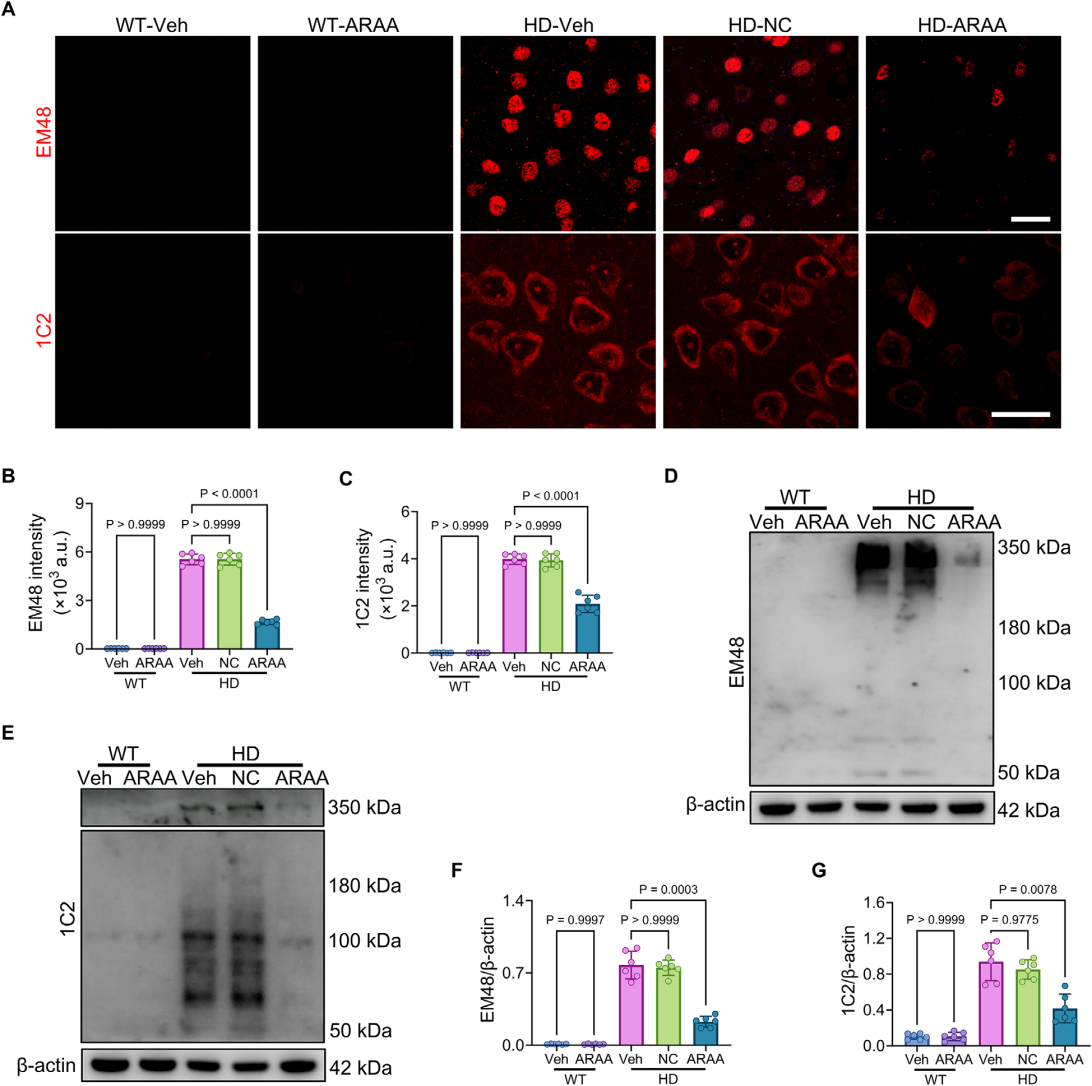
ARAA treatment reduces mHTT aggregate burden in the striatum of R6/2 HD mice. Following behavioral assessments, striatal (STR) sections were analyzed within the following stereotaxic coordinates: AP, +0.0 to +1.0 mm; ML, 0.5–3.0 mm; and DV, 2.0–5.0 mm. (A) Representative immunofluorescence images showing EM48‐positive aggregated mHTT species and 1C2‐positive expanded polyglutamine species in the striatum. EM48 preferentially detects aggregated mHTT species, whereas 1C2 recognizes expanded polyglutamine tracts. Scale bar, 10 µm. (B, C) Quantification of EM48 (B) and 1C2 (C) fluorescence intensities based on the images in (A) using ImageJ software. (D, E) Representative Western blots of striatal homogenates probed with EM48 (D) and 1C2 (E); β‐actin served as a loading control. (F, G) Quantification of EM48‐positive (F) and 1C2‐positive (G) high‐molecular‐weight species based on the blots in (D) and (E), respectively. All data are presented as mean ± SD; n = 6 mice per group; each point represents one mouse. Statistical analysis was performed using one‐way ANOVA, and specific comparisons between groups of interest are indicated. Exact *p* values are shown in the figure unless *p* < 0.0001.

To determine whether the functional improvements observed in R6/2 HD mice were accompanied by reduced mHTT pathology, we examined mHTT burden in the striatum, a principal brain region affected in HD. Immunofluorescence staining revealed abundant EM48‐positive aggregated mHTT and 1C2‐reactive expanded polyQ‐containing species in the striatum of HD‐Veh and HD‐NC mice, whereas both signals were significantly reduced in HD‐ARAA mice (Figure 5A–C). Consistent with the histological findings, Western blot analysis of striatal homogenates showed lower levels of EM48‐positive and 1C2‐positive high‐molecular‐weight species in HD‐ARAA mice than in the HD control groups (Figure 5D–G).

To further assess whether ARAA treatment affected endogenous full‐length HTT abundance, Western blotting of striatal homogenates was performed using MAB2166. Comparable levels of endogenous full‐length HTT were observed across the treatment groups, with no significant difference between WT‐Veh and WT‐ARAA mice (Figure S3C, D). These results suggest that ARAA treatment did not measurably alter endogenous wild‐type HTT abundance under the conditions tested. We next examined whether the reduction in mHTT burden extended beyond the striatum. Consistent with the striatal findings, EM48‐ and 1C2‐positive mHTT signals were also reduced in the cortex of HD‐ARAA mice (Figure S4A–E). Together, these findings indicate that systemic ARAA treatment was associated with reduced mHTT burden in both the striatum and cortex of R6/2 HD mice. In parallel, MAB2166 analysis did not detect a reduction in endogenous full‐length wild‐type HTT abundance under the conditions tested.

**FIGURE 6:**
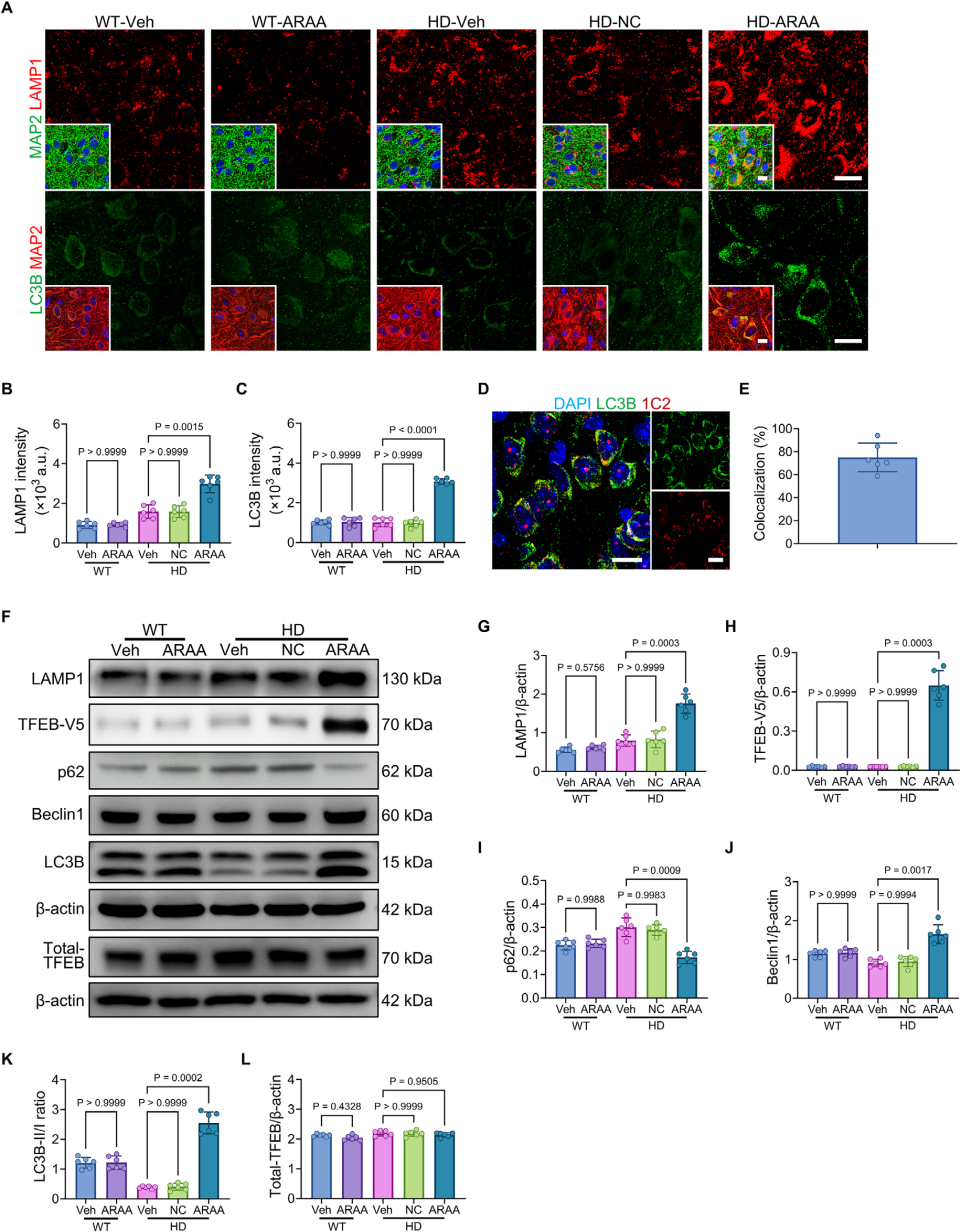
ARAA treatment activates the TFEB‐mediated autophagy–lysosomal pathway in the striatum of R6/2 HD mice. (A) Representative immunofluorescence images showing LAMP1 and LC3B expression in MAP2‐positive neurons in the striatum of mice from the indicated treatment groups. Scale bar, 25 µm. (B, C) Quantification of LAMP1 (B) and LC3B (C) fluorescence intensities based on the images in (A) using ImageJ software. (D) Representative immunofluorescence images showing the colocalization of LC3B with 1C2‐reactive expanded polyQ‐containing mHTT species in the striatum of HD‐ARAA mice. Scale bar, 10 µm. (E) Quantification of LC3B–1C2 colocalization based on the images in (D), calculated as the colocalized area relative to the total 1C2‐positive area using ImageJ software. (F) Representative Western blots showing the protein levels of LAMP1, ARAA‐derived TFEB detected using the V5 tag, total TFEB, p62, Beclin 1, and LC3B in striatal homogenates from the indicated treatment groups; β‐actin served as a loading control. (G–L) Quantification of relative protein levels of LAMP1 (G), V5‐tagged TFEB (H), p62 (I), Beclin 1 (J), the LC3‐II/LC3‐I ratio (K), and total TFEB (L) based on the blots in (F). All data are presented as mean ± SD; *n* = 6 mice per group; each point represents one mouse. Statistical analysis was performed using one‐way ANOVA, and specific comparisons between groups of interest are indicated. Exact *p* values are shown in the figure unless *p* < 0.0001.

Having observed a reduced mHTT burden following ARAA treatment, we next examined autophagy–lysosomal markers in the striatum. Immunofluorescence analysis showed increased LAMP1 staining in MAP2‐positive neurons of HD‐ARAA mice compared with the HD‐Veh and HD‐NC groups. LC3B displayed a predominantly diffuse distribution in the HD control groups, whereas HD‐ARAA mice exhibited increased LC3B intensity and prominent clustered LC3B‐positive structures (Figure 6A–C).

Co‐immunostaining in the HD‐ARAA group showed extensive overlap between LC3B and cytoplasmic 1C2‐reactive expanded polyQ species, with approximately 75% of the 1C2‐positive area colocalizing with LC3B. In contrast, nuclear 1C2‐positive signals showed little overlap with LC3B, consistent with the predominantly cytoplasmic localization of LC3B (Figure 6D,E). These observations indicate a close spatial association between LC3B‐positive structures and a substantial proportion of cytoplasmic expanded polyQ species.

Immunofluorescence analysis showed abundant circuit‐derived V5‐tagged TFEB in the striatum of HD‐ARAA mice, whereas little or no TFEB‐V5 signal was detected in the other groups (Figure S3E,F). Total TFEB staining showed a modest upward trend in the HD‐ARAA group, but no significant differences were observed among the treatment groups (Figure S3E,G). Three‐dimensional reconstruction of MAP2‐positive neurons in HD‐ARAA mice further showed that approximately 74% of the TFEB‐V5 signal was localized within the nucleus, compared with approximately 20% of the total TFEB signal (Figure S3H,I), indicating preferential nuclear localization of the circuit‐derived TFEB pool.

Western blot analysis similarly showed readily detectable V5‐tagged TFEB in the striatum of HD‐ARAA mice, together with increased LAMP1 and Beclin 1 levels, an elevated LC3‐II/LC3‐I ratio, and reduced p62 levels compared with the HD control groups. Total TFEB protein levels remained comparable among groups (Figure 6F–L). Similar changes in V5‐tagged TFEB, p62, and the LC3‐II/LC3‐I ratio were observed in the cortex (Figure S4F–I). Together, these results support an association between systemic ARAA treatment and enhanced TFEB‐related autophagy–lysosomal activity in the striatum and cortex of R6/2 HD mice.

### 2.6 ARAA Alleviates Neurodegenerative Pathology in R6/2 HD Mouse Brains

**FIGURE 7:**
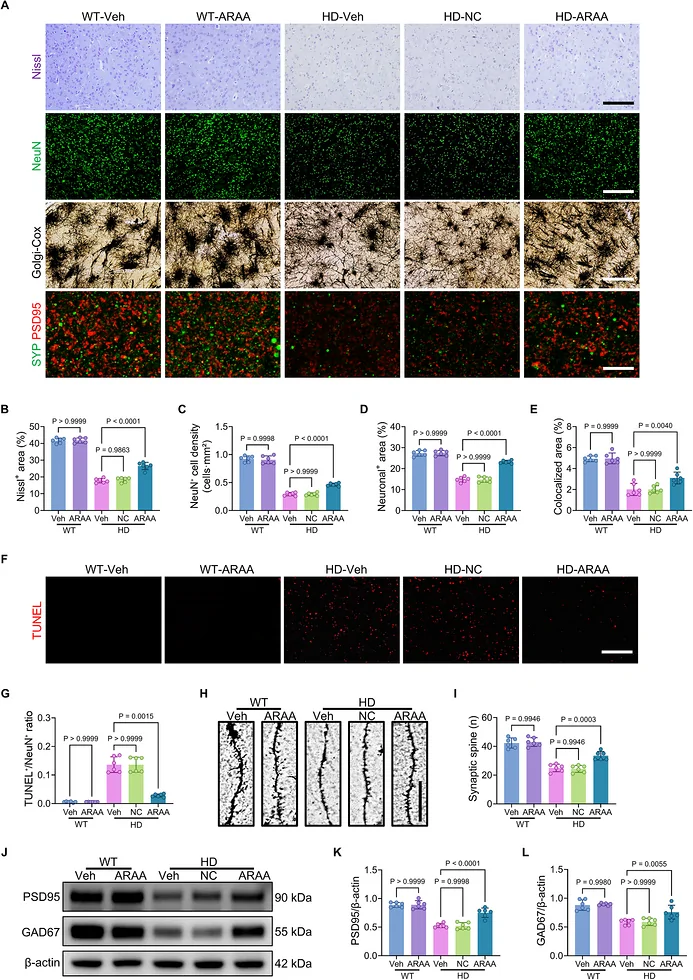
ARAA treatment rescues neuropathological deficits and prevents neuronal loss in the striatum of R6/2 HD mice. (A) Representative striatal sections from the indicated treatment groups (WT‐Veh, WT‐ARAA, HD‐Veh, HD‐NC, HD‐ARAA) analyzed by Nissl staining, NeuN immunofluorescence, Golgi–Cox staining, and co‐immunostaining of PSD95 (red) with synaptophysin (SYP, green). Scale bars: 100 µm (Nissl, NeuN, Golgi) and 5 µm (PSD95/SYP). (B–E) Quantitative analyses of the Nissl‐positive area (B), NeuN‐positive cell density (C), Golgi‐stained neuron density (D), and the PSD95‐SYP colocalization area (E) based on the images in (A) using ImageJ software. (F) Representative immunofluorescence images of TUNEL staining (apoptosis) in the striatum. (G) Quantification of the proportion of TUNEL‐positive cells among NeuN‐labeled neurons from (F) using ImageJ software. (H) Representative images of dendritic spines on striatal neurons. Scale bar, 5 µm. (I) Quantification of dendritic spine density from (H) using ImageJ software. (J) Representative Western blots showing protein levels of PSD95 and GAD67 in striatal homogenates; β‐actin served as a loading control. (K, L) Quantification of PSD95 (K) and GAD67 (L) protein levels based on (J). All data are presented as mean ± SD; *n* = 6 mice per group; each point represents one mouse. Statistical analysis was performed using one‐way ANOVA, and specific comparisons between groups of interest are indicated. Exact *p* values are shown in the figure, except where *p* < 0.0001.

HD is characterized by progressive striatal atrophy, neuronal loss, and synaptic dysfunction [19, 20]. We next examined whether ARAA alleviated these histopathological features. Nissl staining revealed extensive atrophy and loss of Nissl‐positive neurons in HD‐Veh and HD‐NC mice, whereas HD‐ARAA mice exhibited significantly higher neuronal density (Figure 7A,B). Consistently, the density of NeuN‐positive neurons was significantly preserved in the HD‐ARAA group compared with that in controls (Figure 7A,C). To assess structural integrity, we visualized dendritic architecture using Golgi–Cox staining. Neurons in HD‐Veh and HD‐NC mice appeared sparse and poorly arborized; in contrast, HD‐ARAA treatment significantly improved neuronal morphology and increased the density of Golgi‐impregnated neurons (Figure 7A,D). High‐magnification analysis further confirmed a significant increase in dendritic spine density in HD‐ARAA mice (Figure 7H,I). Furthermore, immunohistochemical analysis showed that the levels of PSD95 and synaptophysin (SYP), as well as the extent of intact synapses (quantified as the percentage of PSD95/SYP colocalized area), were significantly enhanced in HD‐ARAA mice relative to HD‐Veh and HD‐NC controls (Figure 7A,E). These findings were corroborated by Western blot analysis (Figure 7J–L). Finally, TUNEL staining revealed widespread neuronal apoptosis in the HD‐Veh and HD‐NC groups, which was significantly reduced by HD‐ARAA treatment (Figure 7F,G). Similar neuroprotective effects were observed in the cortex (Figure S5). Together, these results indicate that ARAA treatment improves neuronal and synaptic integrity in R6/2 HD mice by attenuating atrophy and suppressing apoptosis.

### 2.7 ARAA Attenuates Glial Activation and Neuroinflammation in R6/2 HD Mice

**FIGURE 8:**
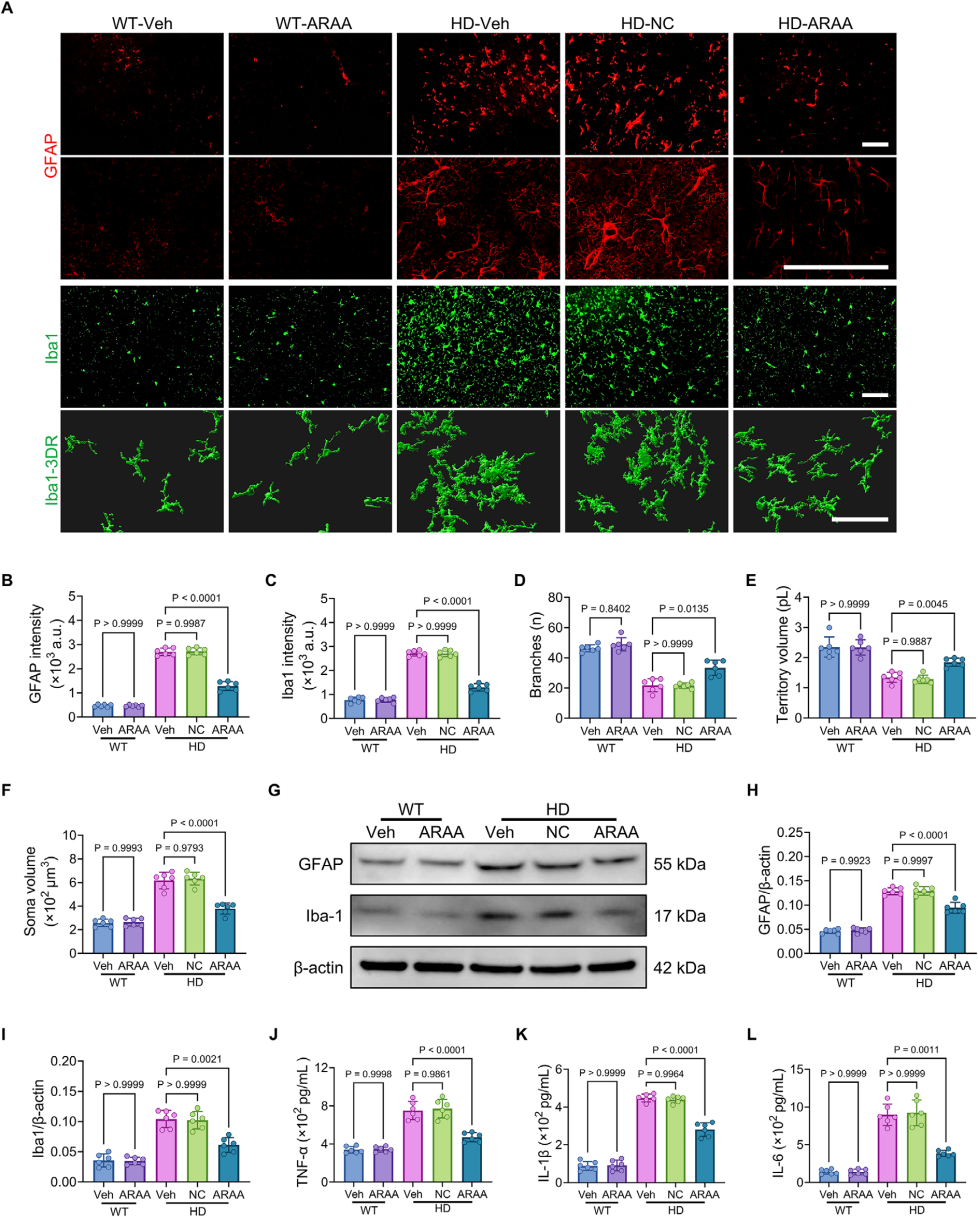
ARAA treatment attenuates neuroinflammation and microglial activation in the striatum of R6/2 HD mice. (A) Representative immunofluorescence images of the striatum from the indicated treatment groups (WT‐Veh, WT‐ARAA, HD‐Veh, HD‐NC, HD‐ARAA), showing astrocytes labeled with GFAP (red) and microglia labeled with Iba1 (green). Representative 3D reconstructions of microglia are shown in the bottom panels. Scale bar, 100 µm. (B, C) Quantification of GFAP (B) and Iba1 (C) fluorescence intensity based on (A) using ImageJ software. (D–F) Morphological analysis of microglia, including the number of branches (D), territory volume (E), and cell body volume (F), was performed on the 3D reconstructions from (A) using Imaris 9.9 software. (G) Representative Western blots showing protein levels of GFAP and Iba1 in striatal tissue; β‐actin was used as a loading control. (H, I) Quantification of relative GFAP (H) and Iba1 (I) protein levels based on (G). (J–L) ELISA measurements of pro‐inflammatory cytokines TNF‐α (J), IL‐1β (K), and IL‐6 (L) in striatal homogenates. All quantitative data are expressed as mean ± SD; *n* = 6 mice per group; each point represents one mouse. Statistical analysis was performed using one‐way ANOVA, and specific comparisons between groups of interest are indicated. Exact *p* values are shown in the figure, except when *p* < 0.0001.

HD pathology is accompanied by reactive gliosis and exacerbated neuroinflammatory responses [20]. We therefore investigated whether ARAA modulated glial activation in vivo. In HD‐Veh and HD‐NC mice, GFAP immunofluorescence was markedly elevated, characterized by hypertrophic astrocytes distributed throughout the neuropil. Likewise, Iba1 staining revealed abundant activated microglia with enlarged somata and shortened, thickened processes (Figure 8A). In contrast, HD‐ARAA mice exhibited visibly reduced GFAP and Iba1 levels, with glial cells displaying morphologies closer to a resting state (Figure 8A–C). Three‐dimensional reconstructions of Iba1‐positive microglia further demonstrated that HD‐ARAA treatment increased branch complexity and territorial arbor volume while reducing soma volume toward a WT‐like morphology (Figure 8A,D–F), indicative of a transition from a reactive to a surveillant phenotype. These findings were corroborated by Western blot analysis (Figure 8G–I). Similar effects were observed in the cortex (Figure S6), suggesting broad suppression of chronic gliosis. Given that glial activation drives pro‐inflammatory cytokine production [20], we measured the levels of TNF‐α, IL‐1β, and IL‐6 in R6/2 HD mouse brains. While these cytokines were markedly elevated in HD‐Veh and HD‐NC mice, HD‐ARAA treatment significantly decreased the levels of all three (Figure 8J–L), consistent with the attenuation of astrocyte and microglial activation.

### 2.8 Therapeutic Mechanism and Translational Considerations

Autophagy dysfunction associated with mHTT accumulation is an important contributor to HD pathogenesis [21]. Current therapeutic strategies face significant hurdles: non‐allele‐selective gene‐silencing approaches can reduce both mutant and wild‐type HTT, whereas broad autophagy activators may impose metabolic stress on healthy cells. To address these limitations, we developed ARAA, a synthetic gene circuit that repurposes the bacterial NarX–NarL system to create a closed‐loop therapeutic device. Unlike constitutive therapies, ARAA links the preferential recognition of aggregated polyQ species by the 11G scFv to conditional TFEB induction. Our biochemical, cellular, Tet‐Off, and in vivo experiments support aggregate‐responsive circuit activation under the conditions tested. In addition, Western blot analysis using MAB2166 showed no significant difference in endogenous full‐length HTT abundance between WT‐Veh and WT‐ARAA mice (Figure S3C,D), indicating that ARAA treatment did not measurably reduce wild‐type HTT protein levels under the present experimental conditions. This finding provides direct experimental support for preservation of steady‐state wild‐type HTT abundance under the conditions tested, although it does not establish that HTT turnover, subcellular localization, or physiological function are unaffected. Nevertheless, the trigger‐responsive design reduced mHTT burden and improved synaptic and behavioral phenotypes while limiting constitutive TFEB activation.

The system's precision relies on the intrinsic signal amplification of the NarX–NarL module and the potent transactivation capability of the VP48 domain [22], which together enable sensitive responses to aggregate‐associated signals. By fusing the NarX sensor with 11G, the circuit exhibited preferential responsiveness to oligomeric polyQ conformers in the assay systems used. The Tet‐Off experiments further showed that circuit‐driven TFEB expression declined as the Q100 burden was reduced, supporting reversible and trigger‐responsive regulation. This behavior may help limit the potential toxicity associated with persistent global TFEB overexpression. Although live‐cell imaging with the tandem mCherry–EGFP–LC3B reporter was used to assess autophagic processing at a defined post‐transfection time point, continuous tracking of aggregate dynamics and circuit‐driven TFEB activation over the full response period was not achieved, as prolonged or repeated imaging of primary neurons introduced phototoxicity and cellular stress. Future studies using lower‐phototoxicity reporters and long‐term imaging platforms will be needed to define the temporal relationship among aggregate accumulation, circuit activation, TFEB induction, and aggregate clearance at the single‐cell level.

The clinical translation of gene circuits is critically limited by the blood‐brain barrier (BBB). While viral vectors are commonly used, they often require invasive stereotactic injections that result in restricted, local distribution. Here, we established a highly effective delivery strategy using CD98hc‐targeted immunoliposomes (LIP‐CD98). CD98hc offers exceptional brain selectivity with minimal lysosomal degradation and low uptake in peripheral organs (such as the liver and spleen) compared to other targets like TfR [16, 23]. By displaying a CD98hc‐binding VNAR on PEGylated liposomes, the formulation supported broad brain distribution of the ARAA plasmid following systemic administration. Expression of the Myc‐tagged sensor was detected predominantly in neurons within the examined cortical and striatal regions, consistent with the use of the hSyn promoter, although limited non‐neuronal expression could not be excluded. Notably, high‐dose safety assessments (three times the therapeutic dose administered every three days for one month) indicated that this non‐viral system was generally well tolerated during the 30‐day dosing period based on the endpoints examined, inducing no overt organ toxicity or significant perturbations in neurodevelopmental or inflammatory gene profiles.

While this study supports the therapeutic potential of the ARAA system, several limitations require further investigation. First, the 30‐day toxicity assessment showed no overt inflammation or organ damage, and intracellular expression of the 11G intrabody may limit systemic exposure to the antibody component. Nevertheless, repeated administration of shark‐derived VNAR proteins and PEGylated liposomes may still induce anti‐drug antibodies, accelerated blood clearance, or tissue accumulation during long‐term treatment. Potential immune responses to the intracellular bacterial NarX–NarL module and synthetic transactivation domain also remain to be evaluated. Accordingly, future studies should include antibody humanization and systematic assessment of long‐term immunogenicity, biodistribution, systemic pharmacokinetics, brain and peripheral‐organ exposure, plasmid persistence, and transgene‐expression duration after repeated dosing. Second, although Western blot analysis using MAB2166 showed comparable endogenous full‐length HTT abundance between WT‐Veh and WT‐ARAA mice, this assessment was limited to steady‐state protein levels. Thus, possible effects of ARAA on wild‐type HTT turnover, subcellular localization, molecular interactions, or physiological function cannot be excluded. This limitation is compounded by the use of the R6/2 model, which reproduces rapid aggregation of the toxic mHTT exon 1 fragment but does not fully reflect the full‐length protein context, proteolytic processing, or gradual adult‐onset progression of human HD. Future work should therefore further assess wild‐type HTT dynamics and HTT‐dependent cellular functions in full‐length or knock‐in models, such as BACHD or zQ175 mice, and in patient‐derived neurons. Finally, only male mice were included to reduce phenotypic variability. Validation in female cohorts will be necessary to determine the robustness and broader applicability of the therapeutic effects.

## 3 Conclusion

In summary, this study presents a programmable gene therapy platform that integrates smart sensing with efficient brain delivery. By coupling the preferential recognition of aggregated polyQ species to the coordinated activation of cellular clearance pathways, ARAA restores proteostasis, alleviates neuroinflammation, and significantly extends survival (from 77 to 102 days) in an R6/2 HD mouse model. Its modular sensor–relay–effector design allows for potential retargeting to other pathological aggregates (e.g., Tau or α‐synuclein), offering a proof‐of‐concept framework for precision medicine in protein aggregation–driven neurodegenerative diseases.

## 4 Experimental Section

### 4.1 ARAA Circuit Design and Plasmid Construction

The ARAA circuit was engineered to conditionally express TFEB in the presence of intracellular mutant huntingtin (mHTT) aggregates. The design comprises three mammalian expression cassettes (pcDNA3.1 backbone): sensor, relay, and effector. The sensor utilizes the 11G scFv, which was isolated from a human naive phage display library (Tomlinson I + J) by screening against GST‐Httex1‐Q53 aggregates, following the conformation‐specific panning protocol described previously [24]. To construct the sensor, the native periplasmic domain of the transmembrane histidine kinase NarX was replaced with 11G, thereby orienting the sensing moiety toward the cytoplasm to detect intracellular polyQ oligomers. The relay is a chimeric transcription factor consisting of the NarL response regulator fused to a VP48 activation domain (VP48–NarL). The effector is mouse TFEB (GenBank NM_011548) bearing a C‐terminal V5 tag. For neuron‐restricted expression, the 11G–NarX sensor and VP48–NarL relay were placed under the human Synapsin I (hSyn) promoter, while TFEB was driven by a synthetic promoter containing tandem NarL‐binding sites upstream of a minimal TA promoter. In the absence of aggregates, NarX and NarL remain inactive, and TFEB expression is minimal. Binding of mHTT oligomers to the cytosolic 11G–NarX is posited to trigger NarX autophosphorylation and subsequent phosphotransfer to NarL, consistent with the established mechanism of the NarX/NarL system [25, 26], which thereby activates the TFEB promoter. A Kozak sequence (GCCACC) was inserted upstream of each start codon. Epitope tags (Myc on 11G–NarX, HA on VP48–NarL, V5 on TFEB) were appended at the C‐termini. All constructs were verified by Sanger sequencing and prepared with EndoFree maxi‐prep kits.

### 4.2 Bacterial Expression of GST‐Httex1‐Q53 and Conformation‐Dependent Recognition Assays

To generate conformation‐controlled antigens, an *E. coli* expression construct encoding the N‐terminal fragment of human Huntingtin (Httex1) with a pathogenic‐range polyQ (Q53) fused to an N‐terminal GST tag was cloned into the pET‐22b (+) vector. The fusion protein (GST‐Httex1‐Q53) was expressed in BL21(DE3) cells induced with 0.2 mm IPTG at 18°C overnight and purified using Glutathione Sepharose 4B (Solarbio, Cat# P2020) affinity chromatography followed by size‐exclusion chromatography (PBS, pH 7.4). To prepare defined antigenic states, the purified fusion protein was divided into two aliquots: one was maintained at 4°C as the monomeric control, where the bulky GST moiety sterically inhibits aggregation; the other was incubated with TEV Protease (Beyotime, Cat# P2301) at 30°C for 4 h to remove the GST tag, thereby triggering the rapid formation of Httex1‐Q53 aggregates [27]. For comparative recognition analysis, samples were spotted onto nitrocellulose membranes for dot blot or coated onto high‐binding plates for ELISA. Membranes were blocked with 5% nonfat‐dried milk and probed with 11G, anti‐GST, or the expanded polyQ‐reactive antibody 1C2 (Millipore, Cat# MAB1574), followed by HRP‐conjugated secondary antibodies and ECL detection (Beyotime, Cat# P0018S). For ELISA, plates coated with monomers or aggregates (2 µg/mL) were blocked with 5% bovine serum albumin, incubated with serial dilutions of 11G, and developed using TMB substrate.

### 4.3 Construction of Mammalian Httex1 (Q6, Q23, and Q100) Expression Plasmids and Dot Blot Analysis

For use as aggregation‐state controls in primary neurons, we constructed mammalian expression plasmids encoding Httex1. Each insert comprised the N17 domain, a variable polyglutamine (polyQ) tract, and the proline‐rich domain. The fragments were cloned into a pcDNA3.1 backbone downstream of a CMV promoter and Kozak sequence. Three variants containing 6, 23, or 100 glutamine residues (Q6, Q23, and Q100, respectively) were generated by gene synthesis and Gibson assembly. Q6 and Q23 were included as short, non‐aggregating controls representing minimal and physiologically non‐expanded polyQ lengths, respectively, whereas Q100 was selected as a strongly aggregation‐prone pathogenic construct for cellular functional studies. A C‐terminal 6×His tag was included for detection in selected experiments. The integrity of the polyQ repeat lengths was verified by bidirectional Sanger sequencing across the flanking regions and by PCR fragment analysis. The plasmids were amplified in *E. coli* Stbl3, and individual clones were screened to exclude repeat‐length alterations. Endotoxin‐free plasmid DNA was prepared for all neuronal transfections.

For dot blot analysis, primary neurons were transfected with the Httex1‐Q6, Httex1‐Q23, or Httex1‐Q100 plasmids as described above. At 3 days after transfection, cells were collected and lysed in a non‐reducing lysis buffer. Lysates were clarified by centrifugation at 13 000 × g, and the protein concentration of the resulting supernatants was determined. An equal amount of total protein (1 µg per spot) was applied to a nitrocellulose membrane. Lysates from untransfected primary neurons were included as the blank control. After air‐drying and blocking, the membranes were separately incubated with the 11G antibody or an anti‐β‐actin antibody, followed by the appropriate horseradish peroxidase‐conjugated secondary antibody. Immunoreactive signals were visualized using an enhanced chemiluminescence detection system. β‐Actin was used as a sample‐loading control.

### 4.4 Lentiviral ARAA and Tetracycline‐Regulated Httex1‐Q100 (Tet‐Off)

For experiments requiring sustained neuronal expression, the three ARAA modules (sensor, relay, effector) were cloned into a self‐inactivating, integration‐competent third‐generation lentiviral backbone (VSV‐G pseudotyped), with the sensor and relay driven by hSyn promoters and TFEB controlled by the NarL‐responsive RETA promoter. Viral particles were produced in HEK293T cells using standard packaging systems and concentrated/filtered per routine procedures; functional titers were determined before use [28]. Neurons were transduced under identical conditions across groups, and investigators were blinded to treatment. Control reagents included empty pcDNA3.1 (negative control, NC) and promoter‐matched reporter vectors to monitor transfection/transduction efficiency. To test circuit reversibility, pathogenic huntingtin was supplied by a tetracycline‐regulated Httex1‐Q100 construct (Tet‐Off). Specifically, a TRE‐tight‐Httex1‐Q100 expression cassette was co‐delivered with a constitutive tTA transactivator driven by an EF1α or PGK promoter. In the absence of doxycycline (doxycycline hyclate, Sigma–Aldrich, Cat# D9891) (−Dox), Httex1‐Q100 is expressed and forms intracellular aggregates; in the presence of doxycycline (+Dox), transcription is silenced, allowing aggregate clearance. Experimental timelines used aggregate “induction” (−Dox) followed by “shutdown” (+Dox) phases to assess ARAA on→off behavior in response to changing aggregate burden, as detailed in the figure legends.

### 4.5 Primary Neuron Culture, Transfection, and Autophagic Flux Analysis

Cortices were isolated from embryonic day 16 C57BL/6 mice, dissociated with 0.15% trypsin at 37°C for 15 min, gently triturated, passed through a 70 µm strainer, and plated on poly‐D‐lysine–coated plates/coverslips in Neurobasal (Thermo Fisher Scientific, Cat# 21103049), supplemented with 2% B‐27 (Thermo Fisher Scientific, Cat# 17504001), 1% GlutaMAX (Thermo Fisher Scientific, Cat# 35050061), and 100 U/mL penicillin–streptomycin (Beyotime, Cat# C0222) [29]. Typical cell seeding densities were 4–5 × 10^5^ cells per well in 6‐well plates or 1 × 10^5^ cells per well in 24‐well plates. Cultures were maintained at 37°C, 5% CO_2_, fed every 3 days, and used at 7–14 DIV. At DIV6–7, neurons were transfected with: (i) Httex1‐Q100 reporter (His tag) and (ii) a single‐vector ARAA plasmid carrying the sensor, relay, and TFEB effector on one backbone (empty vector as NC). Transfections used Lipofectamine 2000 (L2K). Complexes were prepared in Opti‐MEM at a ratio of approximately 2:1 (Lipofectamine 2000: DNA). The typical DNA amount per well was 2–3 µg for 6‐well plates and ∼ 1 µg for 24‐well plates. After 4–6 h, medium was replaced with conditioned Neurobasal. Cells were analyzed at DIV10–11.

For live‐cell assessment of autophagic processing, neurons were transduced with an mCherry–EGFP–LC3B lentiviral reporter (Miaoling Biology, Cat. No. P75170) 3 days before transfection with Httex1‐Q100 and/or ARAA plasmids. At 3 days after plasmid transfection, live neurons were maintained using a stage‐top live‐cell incubation system (Tokai Hit) and imaged with a Leica TCS SP8 confocal microscope. In the tandem reporter assay, mCherry^+^EGFP^+^ yellow puncta were considered autophagosomes, whereas mCherry^+^EGFP^−^ red‐only puncta were considered acidified autolysosomes. Puncta were quantified from the acquired images using ImageJ software.

For autophagic flux analysis, primary neurons were transfected with Httex1‐Q100 and/or ARAA as described above. Three days after plasmid transfection, neurons were treated with bafilomycin A1 (BafA1; Selleck Chemicals, Cat# S1413) or an equivalent volume of vehicle for 4 h immediately before sample collection. Cell lysates were subsequently analyzed by Western blotting for LC3B, p62/SQSTM1, and Beclin 1, with β‐actin used as the loading control. The LC3‐II/LC3‐I ratio and p62 protein levels in the presence and absence of BafA1 were compared to assess autophagic flux. BafA1 was added at a final concentration of 100 nm for 4 h before sample collection.

### 4.6 Tet‐Off Reversibility Assay

For sustained/reversible expression, a subset of cultures at DIV3 was transduced with third‐generation lentiviruses: single‐vector ARAA, in which the sensor and relay were driven by hSyn promoters and TFEB was controlled by the NarL‐responsive RETA promoter, and Tet‐Off Httex1‐Q100 (TRE‐tight cassette plus constitutive tTA driven by an EF1α or PGK promoter). −Dox induced Httex1‐Q100 and aggregate formation; +Dox silenced transcription to permit aggregate clearance. Doxycycline (1 µg/mL) was added at DIV10 where indicated, and TFEB expression and aggregate burden were assessed at defined time points over the following week.

### 4.7 Engineering of Brain‐Targeted Immunoliposomes

We developed a CD98hc‐targeted liposomal carrier to deliver ARAA plasmids to the brain. Cationic liposomes were prepared by thin‐film hydration and extrusion. Briefly, a lipid mixture (DOTAP, cholesterol, DSPE‐PEG2000 at a 49:49:2 molar ratio) was dissolved in chloroform, dried to a thin film under vacuum, and then hydrated in HEPES‐buffered 5% dextrose (pH 7.4) at 65°C for 30 min to yield multilamellar vesicles. These vesicles were sequentially extruded (10–15 passes through 400 and 100 nm polycarbonate membranes) to form uniform unilamellar liposomes (final lipid concentration 5 mg/mL). The anti‐CD98hc single‐domain antibody was derived from a shark VNAR library and enables liposome binding to BBB endothelial CD98hc transporters [16, 30]. The VNAR amino‐acid sequence was obtained from the literature [30], codon‐optimized for *E. coli*, and cloned into a periplasmic expression vector with an OmpA signal peptide and a C‐terminal His‐6 tag. Origami B (DE3) bacteria were transformed and grown in Terrific Broth to OD_600_ ∼1.0, then induced with 0.5 mm IPTG at 27°C for 12 h. The secreted VNAR was purified from culture supernatant by Ni‐NTA affinity chromatography and eluted with imidazole. The His tag was removed by overnight TEV protease digestion, and the cleaved VNAR was passed through Ni‐NTA again to eliminate any uncleaved fraction, yielding highly pure 12 kDa VNAR. To conjugate the nanobody, purified VNAR was thiolated with 2‐iminothiolane (Traut's reagent; Sigma–Aldrich, Cat# I6256; 0.5 mm, 1 h, 25°C) in PBS (pH 7.2), then reacted with DSPE‐PEG2000‐maleimide (Avanti Polar Lipids, Cat# 880126P) at a 1:1.2 molar ratio for 2 h at room temperature to form thioether‐linked conjugates. Residual maleimide was quenched with L‐cysteine (5 mm, 10 min), and products were purified by size‐exclusion chromatography (Sephadex G‐25) [31]. Unreacted VNAR was removed by desalting. The PEGylated VNAR (CD98hc‐targeting moiety) was then inserted into preformed liposomes by mixing at 0.5–1.0 mol% of total lipid and incubating 12 h at 4°C (post‐insertion method). Successful antibody coupling was confirmed by SDS‐PAGE with Coomassie staining, which showed a characteristic band‐shift corresponding to the DSPE‐PEG–VNAR conjugate. These brain‐targeted immunoliposomes (termed LIP‐CD98) were used for all in vivo deliveries of ARAA.

### 4.8 Plasmid DNA Loading Into Liposomes

Endotoxin‐free ARAA plasmid DNA was diluted in HEPES‐buffered 5% glucose and mixed 1:1 (v/v) with the cationic liposome suspension. The nominal N/P ratio was ∼6 (≈10:1 lipid:DNA by mass) unless otherwise specified. Mixtures were incubated for 20–30 min at room temperature to allow charge‐mediated self‐assembly. DNA–liposome complexes were purified by size‐exclusion chromatography on Sepharose CL‐4B, following established liposome SEC workflows [32]. Complexation was confirmed by agarose gel electrophoresis (gel‐retardation). Hydrodynamic diameter, polydispersity index, and Zeta‐potential were determined by dynamic light scattering, and vesicle morphology by transmission electron microscopy. Final immunoliposome formulations were stored at 4°C and used within 1–2 weeks. Endotoxin was quantified by Limulus amebocyte lysate assay and was required to meet predefined acceptance criteria for in vitro and in vivo use.

### 4.9 Liposome Brain Distribution and Neuronal Expression of the ARAA Plasmid

Adult male C57BL/6 mice (8–10 weeks, 22–28 g) received a single lateral tail‐vein injection of Cy5‐labeled, CD98hc‐targeted immunoliposomes (150 µL total) containing ARAA plasmid DNA at 1000 µg/kg, formulated at N/P ≈ 6 (∼ 10:1 lipid:DNA by mass; lipid stock 5 mg/mL; endotoxin met LAL acceptance criteria). At 0, 1, 3, 6, 12, and 24 h post‐dose (n = 3/time point), mice were PBS‐perfused, brains excised, and imaged *ex vivo* on an IVIS (Ex/Em 640/680 nm; exposure/binning/F‐stop/FOV fixed). Whole‐brain ROIs (template‐matched) yielded background‐subtracted radiant efficiency for analysis. A separate cohort was perfused at 48 h for transgene assessment: brains were fixed in 4% PFA (overnight, 4°C), paraffin‐embedded, sectioned (5 µm), and immunostained with anti‐Myc (11G‐NarX), NeuN, and DAPI. Confocal images were acquired on a Leica TCS SP8 under matched laser/detector/pinhole settings; quantitative analyses, when performed, used blinded ROIs on anatomically matched sections.

### 4.10 MTT Cell Viability Assay

*Primary neurons*. Cortical neurons were plated in 96‐well plates (2 × 10^4^ cells/well). Primary cortical neurons were transfected with the indicated plasmids at DIV7, and cell viability was assessed by MTT assay at DIV10. *SH‐SY5Y assay for liposome cytotoxicity*. SH‐SY5Y cells were seeded at 8 × 10^3^ cells/well (96‐well), allowed to adhere overnight, then exposed to immunoliposomes (50 µg/mL, lipid concentration) for 24 h in complete medium. For both assays, MTT (1 mg/mL) was added for 3–4 h at 37°C, supernatant was removed, and formazan was dissolved in DMSO (150 µL/well) with gentle shaking (10 min). Absorbance was read at OD570 with a 630 nm reference on a microplate reader. Viability (%) was calculated relative to matched untreated controls after background subtraction. Each condition was run in technical triplicate; analyses were performed blinded to group.

### 4.11 HD Mouse Model and Treatment Groups

R6/2 Huntington's disease (HD) mice (B6CBA‐Tg (HDexon1) 62Gpb/3J; expressing human *HTT* exon 1 with approximately 150 CAG repeats; The Jackson Laboratory, stock No. 006494) were used as the HD model, and wild‐type (WT) littermates served as controls. Only male mice were included to reduce within‐cohort variability. All procedures were approved by the Institutional Animal Care and Use Committee and complied with national guidelines. Animals were maintained under SPF conditions on a 12‐h light/dark cycle with ad libitum access to food and water. At 4 weeks of age, mice were randomized (n = 8 per group) into HD‐Veh (PBS), HD‐NC (LIP‐CD98 encapsulating empty pcDNA3.1), HD‐ARAA (LIP‐CD98‐ARAA), WT‐Veh (PBS), and WT‐ARAA (LIP‐CD98‐ARAA). Dosing was via lateral tail‐vein injection every 3 days from 4 to 8 weeks. Each injection delivered a total volume of 150 µL containing plasmid at 1000 µg/kg per injection (body‐weight–normalized) and liposome at a lipid:DNA mass ratio of ∼ 10:1; the remainder was sterile PBS to 150 µL. As a reference, for a 25‐g mouse each injection comprised 25 µg plasmid DNA, ∼ 250 µg lipid (e.g., 50 µL of a 5 mg/mL liposome stock), and PBS q.s. to 150 µL. NC animals received empty vector DNA at the same dose; vehicle groups received volume‐matched sterile PBS. Injections were performed with a 27G butterfly needle following brief tail warming, and animals were monitored until full recovery; allocation and assessments were conducted under blinded conditions.

### 4.12 Behavioral Tests and Survival Analysis

*General procedures*. Behavioral assessments were performed during the final 2 weeks of treatment (R6/2 HD mice age 8–10 weeks, when the phenotype is pronounced). Testing occurred in the light phase (ZT4–ZT8) by investigators blinded to genotype and treatment. Mice were acclimated to the testing room for ≥ 30 min before each assay; apparatus were cleaned with 70% ethanol between subjects. *Hindlimb clasping test* [33]. Mice were suspended by the tail for 20 s, and hindlimb posture was scored on a 0–3 scale (0 = no clasp; 3 = sustained full clasp). Each mouse completed three trials separated by ≥ 5 min; the mean score was used as the primary outcome (higher scores indicate worse phenotype). *Rotarod test* [34]. The assay spanned 3 consecutive days. Each day, mice first completed a 300 s adaptation at 4 rpm (training), followed 30 min later by one accelerating trial (4 → 40 rpm over 5 min; 300 s cutoff). For each mouse, the latency to fall from the accelerating trial was recorded daily. *Pole test* [35]. A 50 cm vertical pole (1 cm diameter, rough surface) was used to assess bradykinesia. Mice were placed head‐up near the top. The time to turn completely downward (T‐turn) and the time to descend to the base (T‐descend) were recorded. Each mouse performed three trials (≥ 5 min inter‐trial interval); trials with a fall received a maximum of 20 s. The mean T‐turn and T‐descend were analyzed. *Gait analysis (footprint)* [36]. Hindpaws were dipped in non‐toxic red ink, and mice traversed a paper‐lined runway (50 cm × 10 cm) toward a dark goal box. Clear, sequential footprints were analyzed for stride length and hind base width. Eight consecutive footprint intervals from each mouse were measured and averaged to generate one value per animal. *Wire hang test* [37]. Neuromuscular strength/endurance was measured on a metal wire grid positioned ∼ 40 cm above padding. After grasping the grid, it was gently inverted; latency to fall was recorded with a 60 s cutoff. Three trials were performed with adequate rest; the longest hang time was analyzed. *Open field test* [38]. Spontaneous locomotor activity and anxiety‐like behavior were assessed in a 50 cm × 50 cm open field arena with opaque walls. Each mouse was placed in the center and allowed to explore freely for 5 min. Sessions were recorded and analyzed using the Panlab SMART video tracking system, which quantified the total distance traveled, the time of entries into the center zone (defined as the central 30% of the arena), and rearing events. The primary outcome measures were total distance traveled, the percentage of the total test time spent in the center zone, and the number of rearing events. *Survival analysis*. A separate cohort of mice (*n* = 8 per group) was used exclusively for survival analysis. Mice were monitored daily, and humane endpoints were predefined as a loss of ≥ 20% of peak body weight, inability to eat or drink, or failure to right themselves within 30 s. Survival time was defined as the age at natural death or euthanasia upon reaching a humane endpoint. Assessors remained blinded to group allocation. Survival curves were generated using the Kaplan–Meier method and compared using the log‐rank (Mantel–Cox) test.

### 4.13 Tissue Processing, Histology, and RT‐qPCR Sampling

After completion of all behavioral assessments, at 10 weeks of age, mice were deeply anesthetized with sodium pentobarbital (100 mg/kg, i.p.) and transcardially perfused with ice‐cold PBS. Brains were immediately removed and divided along the midline. The left hemisphere was post‐fixed in 4% PFA at 4°C overnight, embedded in paraffin, and serially sectioned coronally at 5 µm for routine histology and at 20 µm for 3D reconstruction. Serial, anatomically matched sections spanning the striatum and cortex were collected for subsequent staining. The right hemisphere was used for biochemical assays; the striatum and cortex were rapidly dissected on ice, snap‐frozen in liquid nitrogen, and stored at ‐80°C for RNA extraction and RT‐qPCR.

### 4.14 Nissl Staining

Nissl staining was performed to evaluate neuronal viability and cytoarchitecture. Paraffin‐embedded brain sections were deparaffinized, rehydrated through a graded ethanol series, and stained with 0.1% cresyl violet solution (Solarbio, Cat# G1430) for 5 min. Following a brief rinse in distilled water, sections were differentiated in 95% ethanol containing acetic acid, dehydrated in absolute ethanol, cleared in xylene, and mounted with resinous medium. Quantification of Nissl‐positive neurons and the stained neuropil area was conducted in predefined regions of interest, including the striatum and cortical gray matter, using ImageJ. Somatic cross‐sectional area was also measured in striatal neurons from the acquired images to assess cell body size. Six mice were included in each group. One anatomically matched brain section per mouse was analyzed, and three non‐overlapping fields within the predefined region of interest were imaged. Measurements from the three fields were averaged to generate a single value for each mouse, which was used as the unit of statistical analysis.

### 4.15 Golgi‐Cox Staining

A separate therapeutic cohort of mice (*n* = 6 per group) was sacrificed without transcardial perfusion for Golgi–Cox staining. Fresh brains were collected and processed using the FD Rapid GolgiStain Kit (FD NeuroTechnologies, Cat. No. PK401) according to the manufacturer's instructions. Briefly, brains were immersed in equal volumes of Solutions A and B for 2 weeks, transferred to Solution C for 72 h, and sectioned at a thickness of 100 µm. One anatomically matched brain section per mouse was analyzed. For each section, three randomly selected, non‐overlapping fields within the cortex and three within the striatum were imaged and analyzed using ImageJ software. The percentage of Golgi‐positive area was calculated as the proportion of pixels above a constant intensity threshold relative to the total image area. Measurements from the three fields were averaged to generate one value for each brain region of each mouse.

For dendritic spine analysis, 10 well‐impregnated neurons with clearly traceable dendritic arbors were randomly selected from each brain region of each mouse. A 15‐µm segment of a secondary or tertiary dendritic branch was analyzed from each neuron. Dendritic segments with substantial overlap, incomplete impregnation, or obscured branch boundaries were excluded. All visually identifiable protrusions extending from the dendritic shaft were counted as dendritic spines. Spine density was calculated as the number of spines per 15 µm of dendritic length. Values from the 10 neurons were averaged to generate one value for each brain region of each mouse, and the individual mouse was used as the unit of statistical analysis.

### 4.16 TUNEL Assay

Apoptotic cells were identified using the In Situ Cell Death Detection Kit, Fluorescein (Roche, Cat. No. 11684795910) in paraffin‐embedded brain sections and fixed primary neurons. Following permeabilization with ice‐cold 0.1% Triton X‐100 in 0.1% sodium citrate, samples were incubated with the TUNEL reaction mixture containing TdT and fluorescein‐dUTP for 1 h at 37°C in the dark. Nuclei were counterstained with DAPI, and NeuN immunostaining was performed where indicated. Samples were then mounted using an antifade medium.

For brain tissue analysis, six mice were included in each group. One anatomically matched brain section per mouse was analyzed, and three randomly selected, non‐overlapping fields within the predefined cortical or striatal region of interest were imaged. Apoptosis was quantified as the percentage of TUNEL‐positive nuclei among NeuN‐positive neurons. Values from the three fields were averaged to generate one value for each mouse, and the individual mouse was used as the unit of statistical analysis.

For cultured‐neuron analysis, three randomly selected, non‐overlapping fields were analyzed for each independent culture. Apoptosis was quantified as the percentage of TUNEL‐positive nuclei among total DAPI‐positive nuclei in each field. Values from the three fields were averaged to generate one value for each independent culture, which was used as the unit of statistical analysis.

### 4.17 Immunofluorescence Staining, Imaging, and Quantitative Analysis

#### 4.17.1 Cultured Neurons

Cells grown on coverslips were fixed with 4% paraformaldehyde for 20 min at room temperature, permeabilized with 0.1–0.2% Triton X‐100 in PBS for 10 min, and blocked with 5%–10% normal donkey serum for 1 h. Primary antibodies diluted in blocking buffer were applied overnight at 4°C. The primary antibodies included mouse anti‐His tag (Abcam, Cat. No. ab18184; 1:500), rabbit anti‐Myc tag (Abcam, Cat. No. ab32072; 1:200), rabbit anti‐HA tag (Abcam, Cat. No. ab236632; 1:200), mouse anti‐V5 tag (Abcam, Cat. No. ab27671; 1:100), chicken anti‐MAP2 (Thermo Fisher Scientific, Cat. No. PA1‐10005; 1:1000), and rabbit anti‐PSD95 (Abcam, Cat. No. ab238135; 1:200), together with other antibodies as indicated. After washing, cells were incubated with species‐appropriate Alexa Fluor 488‐, 546‐, or 647‐conjugated secondary antibodies (Thermo Fisher Scientific; 1:500) for 1 h at room temperature. Nuclei were counterstained with DAPI, and the coverslips were mounted using an antifade mounting medium.

#### 4.17.2 Brain Sections

Immunofluorescence staining was performed on deparaffinized brain sections. Where indicated, antigen retrieval was conducted in 10 mm citrate buffer (pH 6.0) at 95°C for 20 min. Sections were permeabilized with 0.2% Triton X‐100, blocked with 5% normal goat or donkey serum for 1 h, and incubated with primary antibodies overnight at 4°C. The primary antibodies included rabbit anti‐Myc tag (Abcam, Cat. No. ab32072; 1:200), mouse anti‐V5 tag (Abcam, Cat. No. ab27671; 1:100), chicken anti‐MAP2 (Thermo Fisher Scientific, Cat. No. PA1‐10005; 1:1000), mouse anti‐huntingtin EM48 (Sigma–Aldrich, Cat. No. MAB5374; 1:200), rabbit anti‐LC3B (Cell Signaling Technology, Cat. No. 83506; 1:200), mouse anti‐NeuN (Beyotime, Cat. No. AG5317; 1:500), rabbit anti‐PSD95 (Abcam, Cat. No. ab238135; 1:200), mouse anti‐synaptophysin (SYP; Abcam, Cat. No. ab8049; 1:500), mouse anti‐GAD67 (Abcam, Cat. No. ab26116; 1:200), mouse anti‐GFAP (Cell Signaling Technology, Cat. No. 3670; 1:500), rabbit anti‐TFEB (Cell Signaling Technology, Cat. No. 83010T; 1:100), rabbit anti‐LAMP1 (Abcam, Cat. No. ab208943; 1:100), rabbit anti‐Iba1 (GeneTex, Cat. No. GTX100042; 1:500), mouse anti‐Olig2 (Cell Signaling Technology, Cat. No. 39588T; 1:200), and mouse anti‐expanded polyQ antibody 1C2 (Millipore, Cat. No. MAB1574; 1:1000). After washing, sections were incubated with species‐appropriate Alexa Fluor‐conjugated secondary antibodies (Thermo Fisher Scientific; 1:500) for 2 h at room temperature, followed by DAPI counterstaining and mounting with an antifade mounting medium.

#### 4.17.3 Imaging and Quantification

Fluorescence images were acquired using a Leica TCS SP8 confocal microscope with identical laser power, detector gain, pinhole size, and other acquisition settings across groups within each experiment. Bright‐field images, including H&E, chromogenic immunohistochemistry, and Golgi–Cox staining, were acquired using an Olympus IX73 microscope. Unless otherwise specified, one anatomically matched brain section per mouse and three non‐overlapping fields within the predefined region of interest were analyzed. Image selection and quantification were performed using ImageJ by an investigator blinded to treatment allocation.

EM48‐positive mHTT burden was quantified as the positive inclusion area normalized to the total region‐of‐interest area or DAPI‐positive cell number, as indicated. NeuN‐positive cell density was determined within defined cortical and striatal regions and expressed as cells/mm^2^. GFAP and Iba1 fluorescence intensities were quantified using equivalent regions of interest and consistent thresholds across groups.

For synaptic colocalization, 20‐µm‐thick sections were co‐stained for PSD95 and synaptophysin. Confocal z‐stacks were acquired at 1‐µm intervals and projected using identical settings. Following background subtraction and consistent thresholding, colocalization was defined as the overlapping area of the PSD95‐ and synaptophysin‐positive masks and expressed as a percentage of the analyzed region. Values from three fields were averaged to generate one value per mouse.

For three‐dimensional microglial analysis, clearly distinguishable Iba1‐positive cells that were fully contained within the confocal z‐stack were reconstructed using Imaris 9.9. The Filaments and Surfaces modules were used to quantify branch parameters, soma volume, and cell volume under identical reconstruction criteria across all groups. At least 10 microglial cells were analyzed from each mouse. Cell‐level measurements were first averaged within each mouse to generate a single biological replicate. Six mice were included in each group, and the individual mouse, rather than the individual cell, was used as the unit of statistical analysis.

### 4.18 Western Blotting and Biochemical Analysis

For cultured primary neurons, cells were washed twice with ice‐cold PBS and lysed directly in the culture plates by adding ice‐cold RIPA buffer containing 50 mm Tris‐HCl (pH 7.4), 150 mm NaCl, 1% NP‐40, 0.5% sodium deoxycholate, and 0.1% SDS, supplemented with protease and phosphatase inhibitor cocktails. The cells were lysed on ice, and the lysates were collected by repeated pipetting and transferred into prechilled tubes.

For brain tissue analysis, the striatum and cortex were rapidly dissected on ice and separately homogenized in the same ice‐cold RIPA buffer supplemented with protease and phosphatase inhibitors. A consistent tissue‐to‐buffer ratio was maintained across all samples. Cell lysates and tissue homogenates were centrifuged at 15 000 × g for 20 min at 4°C, and the resulting supernatants were collected as total protein lysates.

Protein concentrations were determined using a bicinchoninic acid assay. Equal amounts of protein, typically 20 µg per lane, were mixed with Laemmli sample buffer containing β‐mercaptoethanol, heated at 95°C for 5 min, separated using 4%–20% gradient precast SDS–PAGE gels, and transferred onto 0.45 µm NC membranes. Membranes were blocked with 5% non‐fat milk in Tris‐buffered saline containing 0.1% Tween‐20 (TBST) for 1 h at room temperature and then incubated overnight at 4°C with the following primary antibodies: mouse anti‐His tag (Abcam, Cat. No. ab18184, 1:500); rabbit anti‐Myc tag (Abcam, Cat. No. ab32072, 1:200); rabbit anti‐HA tag (Abcam, Cat. No. ab236632, 1:200); mouse anti‐V5 tag (Abcam, Cat. No. ab27671, 1:100); mouse anti‐mHTT aggregate antibody EM48 (Sigma–Aldrich, Cat. No. MAB5374, 1:1000); mouse anti‐polyQ antibody 1C2 (Millipore, Cat. No. MAB1574, 1:1000); mouse anti‐Huntingtin antibody (Sigma–Aldrich, Cat. No. MAB2166, 1:1000); rabbit anti‐LC3B (Cell Signaling Technology, Cat. No. 83506, 1:1000); rabbit anti‐p62/SQSTM1 (Beyotime, Cat. No. AF5312, 1:500); rabbit anti‐PSD95 (Abcam, Cat. No. ab238135, 1:1000); mouse anti‐GAD67 (Abcam, Cat. No. ab26116, 1:1000); mouse anti‐GFAP (Cell Signaling Technology, Cat. No. 3670, 1:2000); rabbit anti‐Iba1 (GeneTex, Cat. No. GTX100042, 1:1000); rabbit anti‐TFEB (Cell Signaling Technology, Cat. No. 83010T, 1:1000); rabbit anti‐Beclin1 (Beyotime, Cat. No. AF5123, 1:1000); rabbit anti‐LAMP1 (Abcam, Cat. No. ab208943, 1:1000); and mouse anti‐β‐actin (Proteintech, Cat. No. 66009‐1‐Ig, 1:5000), which was used as the loading control.

After three washes with TBST, membranes were incubated with the appropriate HRP‐conjugated secondary antibodies at a dilution of 1:5000 for 1 h at room temperature. Protein signals were developed using an enhanced chemiluminescence substrate and captured with a digital chemiluminescence imaging system. Band intensities were quantified using ImageJ, normalized to β‐actin, and expressed relative to the mean value of the corresponding control group on each blot.

### 4.19 Enzyme‐Linked Immunosorbent Assay (ELISA)

Proinflammatory cytokines TNF‐α, IL‐1β, and IL‐6 were quantified in brain homogenates (striatum and cortex). Tissue was homogenized in ice‐cold PBS containing 1% Triton X‐100 and protease inhibitors, cleared at 12 000 × g for 10 min at 4°C, and supernatants were assayed using multiplex ELISA kits (Biolegend) per the manufacturer's protocol. Plates were read at OD_450_ with a 570/620 nm reference, and concentrations were interpolated from standard curves (4‐parameter logistic fit). Values were normalized to total protein (BCA). Samples were run in duplicate, with the experimenter blinded to group.

### 4.20 Subchronic Dosing and Tissue Collection (Toxicity Cohort)

At 4 weeks of age, male wild‐type C57BL/6 mice (*n* = 6) were administered LIP‐CD98‐ARAA at a high dose of 3000 µg plasmid DNA/kg (three times the therapeutic dose) via tail vein injection once every three days over a period of 30 days. The injection volume was maintained at ≤ 300 µL per mouse. Animals were monitored daily for clinical signs and body weight. On day 31, mice were transcardially perfused with ice‐cold PBS under deep anesthesia. Major organs (heart, liver, spleen, lung, kidney) and the left hemisphere of the brain were harvested, fixed in 4% paraformaldehyde (overnight at 4°C), paraffin‐embedded, and sectioned at 5 µm. Sections were stained using an H&E staining kit (Solarbio) according to the manufacturer's instructions. The entire right hemisphere was immediately isolated, snap‐frozen in liquid nitrogen, and stored at −80°C for total RNA extraction and RT‐qPCR analysis.

### 4.21 RT‐qPCR

Total RNA was extracted from cultured neurons or frozen cortex/striatum (TRIzol or equivalent column kit with on‐column DNase). cDNA was synthesized from 1 µg RNA (High‐Capacity Reverse Transcription kit or equivalent). RT‐qPCR was run on a CFX96 (Bio‐Rad) using SYBR Green Master Mix in technical triplicate. Primers targeted TFEB/CLEAR genes (*Wipi1*, *Mcoln1*, *Ctsd*, *Atp6v1a*, *Gabarapl1*) and selected markers (e.g., *Bdnf*, cytokines); sequences were designed from published references or validated databases and are listed in Table S1. β‐actin (*Actb*) served as the housekeeping gene. Ct values were analyzed by the 2^−ΔΔCt^ method with appropriate calibration controls. Results were expressed as relative expression (fold‐change vs. control). Runs included no‐template and no‐RT controls; analyses were performed blinded to group.

### 4.22 Statistical Analysis

Data preprocessing and normalization were performed as described for each assay. Western blot signals were normalized to the corresponding loading control and expressed relative to the indicated control group. RT‐qPCR data were analyzed using the 2^−ΔΔCt^ method, and imaging measurements were aggregated as described in the corresponding experimental sections. No additional data transformation was applied unless otherwise specified. No data points were excluded solely on the basis of statistical outlier testing; samples were excluded only in cases of predefined technical failure.

Data are presented as mean ± SD. Sample sizes are specified in the corresponding figure legends. For cultured‐cell experiments, *n* represents biologically independent cultures or experiments, whereas for animal studies, *n* represents individual mice. For imaging‐based animal experiments, measurements from multiple fields or cells were averaged to generate one value per mouse, and the individual mouse was used as the unit of statistical analysis.

Statistical analyses were performed using GraphPad Prism 9. Data distributions were assessed using the Shapiro–Wilk test, and homogeneity of variance was evaluated using the Brown–Forsythe or Levene's test, as appropriate. Comparisons between two groups were performed using an unpaired two‐tailed Student's *t*‐test when the assumptions of normality and equal variance were satisfied; Welch's *t*‐test was used when variances were unequal. Multiple‐group comparisons were performed using one‐way or two‐way ANOVA, as appropriate. Tukey's multiple‐comparisons test was used when variances were homogeneous, whereas Welch's ANOVA followed by Dunnett's T3 or Games–Howell post hoc test was used when the assumption of equal variance was not satisfied. Repeated‐measures behavioral data were analyzed using two‐way repeated‐measures ANOVA with treatment or genotype and trial or time as factors, followed by the post hoc test specified in the corresponding figure legend. Survival curves were compared using the two‐sided log‐rank (Mantel–Cox) test. All statistical tests were two‐sided, and *p* < 0.05 was considered statistically significant. Exact *p* values are reported in the figures or figure legends, except when *p* < 0.0001.

## Author Contributions

J. Zhu, R.‐t. Liu, and G.‐f. Zhang conceived and designed the project. J. Zhu, X.‐x. Xie, and L. Li performed the experiments and acquired the data. C. Tian, H.‐t. Wang, and X.‐j. Wang assisted with the animal experiments and biochemical assays. X.‐l. Yu performed the formal analysis and data curation. J. Zhu wrote the original draft. G.‐f. Zhang and R.‐t. Liu reviewed and edited the manuscript. G.‐f. Zhang and R.‐t. Liu provided supervision and funding acquisition. All authors have read and agreed to the published version of the manuscript.

## Ethics Statement

All animal procedures complied with the *Guide for the Care and Use of Laboratory Animals* and ARRIVE guidelines. The protocol was approved by the Institutional Animal Care and Use Committee (IACUC) of the Institute of Process Engineering, Chinese Academy of Sciences (Approval code: IPEAECA2024074; Approval date: 9 December 2024).

## Conflicts of Interest

The authors declare no conflicts of interest.

## Supporting information




**Supporting File**: advs78018‐sup‐0001‐SuppMat.docx.

## Data Availability

The data that support the findings of this study are available from the corresponding author upon reasonable request.
