# Supplementary material for: Huntingtin Aggregate‐Responsive Autophagy Gene Circuit Mitigates Disease Pathology in R6/2 Mice
Source: Adv Sci (Weinh). 2026 Sep 27:e78018. Online ahead of print. doi: 10.1002/advs.78018 (PMC13616256; doi:10.1002/advs.78018)
Supplement: Supplementary file 1 [file ADVS-9999-e78018-s001.docx]

**Huntingtin Aggregate-Responsive Autophagy Gene Circuit Mitigates Disease Pathology in R6/2 Mice**

Jie Zhu^1,2^ †, Xi-xiu Xie^1^ †, Lei Li^3^ †, Chen Tian^1,2^, Hao-tian Wang^1,2^, Xiao-jie Wang^1,2^, Xiao-lin Yu^1^, Gui-feng Zhang^1,*^, Rui-tian Liu^1,*^

**Affiliations**

^1^ National Key Laboratory of Biopharmaceutical Preparation and Delivery, Institute of Process Engineering, Chinese Academy of Sciences, Beijing 100190, China

^2^ University of Chinese Academy of Sciences, Beijing 100049, China

^3^ Department of Obstetrics and Gynecology, National Clinical Research Center for Obstetric & Gynecologic Diseases, Peking Union Medical College Hospital, Chinese Academy of Medical Sciences and Peking Union Medical College

***Corresponding author**

Rui-tian Liu, Gui-feng Zhang

National Key Laboratory of Biopharmaceutical Preparation and Delivery, Institute of Process Engineering, Chinese Academy of Sciences, Haidian District, Beijing 100190, China, Tel.: +86 10 82545017; Fax: +86 10 82544904; E-mail: [rtliu@ipe.ac.cn](mailto:rtliu@ipe.ac.cn)

† These authors contributed equally to this work.

**Table of Contents**

**Figure S1.** Characterization of the aggregate-recognizing 11G scFv, ARAA component expression, and autophagy-related responses in primary neurons. **Figure S2.** Reversible activation of the ARAA circuit in a Tet-Off system. **Figure S3.** Cellular distribution of 11G–NarX, endogenous full-length HTT levels, and expression and nuclear localization of ARAA-derived TFEB in the striatum. **Figure S4.** ARAA reduces mHTT burden and enhances autophagy–lysosomal responses in the cortex. **Figure S5.** ARAA ameliorates neuropathological deficits in the cortex. **Figure S6.** ARAA attenuates neuroinflammation in the cortex. Table S1. List of primers used for RT-qPCR.

**Figure S1.** **Characterization of the aggregate-recognizing 11G scFv, expression of ARAA components, and assessment of autophagy-related responses in primary neurons.** **(A)** Dot blot analysis of 11G binding to GST, GST-Httex1-Q53 (fusion protein), and Httex1-Q53 aggregates (GST cleaved). 1C2 (anti-polyQ) and anti-GST antibodies served as controls. **(B)** ELISA measuring the binding affinity of Myc-tagged 11G to Httex1-Q53 aggregates. Serial two-fold dilutions starting from 20 μg/mL were used. **(C)** Dot blot analysis of 11G binding to protein homogenates from primary neurons expressing Httex1-Q6, Q23, or Q100. β-actin served as a loading control. **(D)** Native-PAGE immunoblot of polyQ species immunoprecipitated by 11G from WT and R6/2 mouse brain homogenates, detected using the anti-polyQ antibody 1C2 (MAB1574). **(E)** Representative immunofluorescence images showing the expression of the ARAA sensor and relay modules in primary neurons. **(F)** Quantification of transfection efficiency based on **(E)** (calculated as the percentage of MAP2-positive neurons expressing the constructs; n = 3 biologically independent replicates). **(G)** Representative immunofluorescence images confirming the expression and localization of the ARAA sensor (11G–NarX) and relay (VP48–NarL) in MAP2-positive (red) primary neurons expressing ARAA alone or co-expressing ARAA with Httex1-Q100. Scale bar, 100 μm. **(H, I)** Quantification of fluorescence intensity from **(G)** using ImageJ software. **(J)** Representative Western blots showing the expression of the ARAA sensor and relay in the indicated treatment groups; β-actin served as a loading control. **(K, L)** Quantification of 11G-NarX **(K)** and VP48-NarL **(L)** protein levels based on **(J)**. **(M)** Representative Western blots showing the levels of autophagy-related proteins in primary cortical neurons expressing Q100 alone or co-expressing Q100 and ARAA, in the presence or absence of bafilomycin A1 (BafA1) to block autophagic flux; β-actin served as a loading control. **(N–P)** Quantification of p62 **(N)**, Beclin 1 **(O)**, and the LC3-II/LC3-I ratio **(P)** based on the blots in (M). All data are presented as mean ± SD; n = 6 biologically independent replicates unless otherwise noted. Statistical analysis was performed using one-way ANOVA, and specific comparisons between groups of interest are indicated. Exact *P* values are shown in the figure unless *P* < 0.0001.

**Figure S2. ARAA-driven TFEB expression is reversible and returns to a dormant state following polyQ suppression in a Tet-Off system.** **(A)** Schematic illustration of the Tet-Off experimental design. Primary cortical neurons were co-transduced with LV-Httex1-Q100 (Tet-Off) and LV-ARAA at DIV3. At DIV10, doxycycline (Dox; 1 μg/mL) was added to suppress de novo Q100 expression, and ARAA-driven TFEB expression was monitored over the subsequent week. **(B)** Representative immunofluorescence images of TFEB (V5, green) in MAP2-positive (red) neurons. Scale bar, 10 μm. **(C)** Quantification of TFEB fluorescence intensity from **(B)** using ImageJ software. **(D)** Western blot analysis of TFEB (V5) protein levels; β-actin served as a loading control. **(E)** Quantification of relative TFEB expression based on **(D)**. **(F)** Dot blot analysis of temporal changes in 1C2-reactive expanded polyQ species during the experimental period; β-actin served as a loading control. **(G)** Quantification of 1C2-reactive expanded polyQ levels based on the dot blot results in **(F)**. All data are presented as mean ± SD; n = 6 biologically independent replicates. Statistical analysis was performed using two-way ANOVA. Exact *P* values are shown in the figure unless *P* < 0.0001.

**Figure S3. Cellular distribution of 11G–NarX, endogenous full-length HTT levels, and expression and nuclear localization of ARAA-derived TFEB in the striatum of R6/2 HD mice. (A)** Representative immunofluorescence images showing the colocalization of the ARAA sensor 11G–NarX with the neuronal markers MAP2 and NeuN, the oligodendrocyte marker Olig-2, the microglial marker Iba1, and the astrocyte marker GFAP in the striatum of HD-ARAA mice. Scale bar, 50 μm. **(B)** Quantification of the percentage of 11G–NarX-positive cells colabeled with each indicated cell-type marker based on the images in **(A)**. **(C)** Representative Western blot analysis of endogenous full-length HTT in striatal homogenates from mice in the indicated treatment groups using MAB2166; β-actin served as a loading control. **(D)** Quantification of endogenous full-length HTT protein levels based on the blots in **(C)**. **(E)** Representative immunofluorescence images showing the expression of ARAA-derived TFEB, detected using the V5 tag (TFEB-V5), in GAD67-positive striatal neurons, and total TFEB in mice from the indicated treatment groups. Scale bars, 10 μm for TFEB-V5 and 25 μm for total TFEB. **(F, G)** Quantification of TFEB-V5 **(F)** and total TFEB **(G)** fluorescence intensities based on the images in **(E)** using ImageJ software. **(H)** Representative three-dimensional reconstructions of immunofluorescence images showing the nuclear localization of TFEB-V5 or total TFEB in MAP2-positive striatal neurons of HD-ARAA mice. Nuclei were counterstained with Hoechst. Scale bar, 10 μm. **(I)** Quantification of the nuclear localization of TFEB-V5 and total TFEB based on the images in **(H)**, calculated as the percentage of nuclear TFEB fluorescence intensity relative to the corresponding total cellular TFEB fluorescence intensity. All data are presented as mean ± SD; n = 6 mice per group; each point represents one mouse. Statistical analysis was performed using one-way ANOVA, and specific comparisons between groups of interest are indicated. Exact P values are shown in the figure unless P < 0.0001.

**Figure S4. ARAA reduces mHTT burden and enhances autophagy–lysosomal responses in the cortex.** Cortical sections from the indicated treatment groups were analyzed. **(A)** Representative immunofluorescence images showing EM48-positive aggregated mHTT species (red) in the cortex. Scale bar, 10 μm. **(B)** Quantification of EM48 fluorescence intensity based on the images in **(A)** using ImageJ software. **(C)** Representative Western blots of cortical homogenates probed with EM48 and 1C2 to detect aggregated mHTT and expanded polyQ-containing species, respectively; β-actin served as a loading control. **(D, E)** Quantification of EM48-positive **(D)** and 1C2-positive **(E)** high-molecular-weight species based on the blots in **(C)**. **(F)** Representative Western blots of autophagy-related proteins in cortical homogenates; β-actin was used as a loading control. **(G–I)** Quantification of protein levels of TFEB (detected via V5 tag) **(G)**, the LC3B-II/LC3B-I ratio **(H)**, and p62 **(I)** based on **(F)**. All data are presented as mean ± SD; n = 6 mice per group; each point represents one mouse. Statistical analysis was performed using one-way ANOVA, and specific comparisons between groups of interest are indicated. Exact *P* values are shown in the figure unless *P* < 0.0001.

**Figure S5. ARAA ameliorates neuropathological deficits in the cortex of R6/2 HD mice.** **(A)** Representative cortical sections from the indicated treatment groups (WT-Veh, WT-ARAA, HD-Veh, HD-NC, HD-ARAA) analyzed by Nissl staining, NeuN immunofluorescence, Golgi–Cox staining, and TUNEL staining (apoptosis). Scale bar, 100 μm. **(B–E)** Quantitative analyses of the Nissl-positive area **(B)**, NeuN-positive cell density **(C)**, Golgi-stained neuron density **(D)**, and the proportion of TUNEL-positive neurons **(E)** based on the images in **(A)** using ImageJ software. **(F)** Representative images of dendritic spines in cortical neurons. Scale bar, 5 μm. **(G)** Quantification of dendritic spine density from **(F)** using ImageJ software. **(H)** Representative Western blots showing protein levels of PSD95 and GAD67 in cortical homogenates; β-actin served as a loading control. **(I, J)** Quantification of PSD95 **(I)** and GAD67 **(J)** protein levels based on **(H)**. All data are presented as mean ± SD; n = 6 mice per group; each point represents one mouse. Statistical analysis was performed using one-way ANOVA, and specific comparisons between groups of interest are indicated. Exact *P* values are shown in the figure, except where *P* < 0.0001.

**Figure S6. ARAA treatment attenuates neuroinflammation in the cortex of R6/2 HD mice.** **(A)** Representative immunofluorescence images of the cortex from the indicated treatment groups (WT-Veh, WT-ARAA, HD-Veh, HD-NC, HD-ARAA), showing astrocytes labeled with GFAP (red) and microglia labeled with Iba1 (green). Scale bar, 100 μm. **(B, C)** Quantification of GFAP **(B)** and Iba1 **(C)** fluorescence intensity based on **(A)** using ImageJ software. **(D)** Representative Western blots showing protein levels of GFAP and Iba1 in cortical homogenates; β-actin was used as a loading control. **(E, F)** Quantification of GFAP **(E)** and Iba1 **(F)** protein levels based on **(D)**. All data are presented as mean ± SD; n = 6 mice per group; each point represents one mouse. Statistical analysis was performed using one-way ANOVA, and specific comparisons between groups of interest are indicated. Exact *P* values are shown in the figure, except where *P* < 0.0001.

**Table S1. List of primers used for RT-qPCR**

| **Gene Name** | **Forward Primer Sequence (5'-3')** | **Reverse Primer Sequence (5'-3')** |
| --- | --- | --- |
| ***Ntn1*** | **GTCTGGTGTGTGACTGTAGGCA** | **CCGAGCATGGAGGTTGCAGTTG** |
| ***Hspa1a*** | **ACAAGTCGGAGAACGTGCAGGA** | **GTTGTCCGAGTAGGTGGTGAAG** |
| ***Wipi1*** | **GAGTTCTGTGGAGCAGCTTGAC** | **GACGTTCATCTGCCGAGGTTTTG** |
| ***Mcoln1*** | **GTCGGTGTCATTCGCTACCTGA** | **GAACGATCCAGCCACAGAAGCA** |
| ***Ctsd*** | **TAAGACCACGGAGCCAGTGTCA** | **CCACAGGTTAGAGGAGCCAGTA** |
| ***Atp6v1a*** | **GCTGGCTTCTTTCTATGAGCGAG** | **GCGTTGCAGAAGTGACTGGATC** |
| ***Gabarapl1*** | **GTGGAGAAGGCTCCTAAAGCCA** | **AGGTCTCAGGTGGATCCTCTTC** |
| ***Trp53*** | **CACAGCACATGACGGAGGTC** | **TCCTTCCACCCGGATAAGATG** |
| ***Syp*** | **TTGGCTTCGTGAAGGTGCTGCA** | **ACTCTCCGTCTTGTTGGCACAC** |
| ***Ifng*** | **CAGCAACAGCAAGGCGAAAAAGG** | **TTTCCGCTTCCTGAGGCTGGAT** |
| ***Bax*** | **AGGATGCGTCCACCAAGAAGCT** | **TCCGTGTCCACGTCAGCAATCA** |
| ***Th*** | **TGCACACAGTACATCCGTCATGC** | **GCAAATGTGCGGTCAGCCAACA** |
| ***Casp3*** | **GGAGTCTGACTGGAAAGCCGAA** | **CTTCTGGCAAGCCATCTCCTCA** |
| ***Sod1*** | **GGTGAACCAGTTGTGTTGTCAGG** | **ATGAGGTCCTGCACTGGTACAG** |
| ***Tfeb*** | **CGCCTGGAGATGACTAACAAGC** | **GGCAACTCTTGCTTCACCACCT** |
| ***Foxp3*** | **CCTGGTTGTGAGAAGGTCTTCG** | **TGCTCCAGAGACTGCACCACTT** |
| ***Gad1*** | **CGCTTGGCTTTGGAACCGACAA** | **GAATGCTCCGTAAACAGTCGTGC** |
| ***Mbp*** | **ATTCACCGAGGAGAGGCTGGAA** | **TGTGTGCTTGGAGTCTGTCACC** |
| ***Slc2a1*** | **GCTTCTCCAACTGGACCTCAAAC** | **ACGAGGAGCACCGTGAAGATGA** |
| ***Bdnf*** | **GGCTGACACTTTTGAGCACGTC** | **CTCCAAAGGCACTTGACTGCTG** |
| ***Tnf-α*** | **GGTGCCTATGTCTCAGCCTCTT** | **GCCATAGAACTGATGAGAGGGAG** |
| ***Il-6*** | **TACCACTTCACAAGTCGGAGGC** | **CTGCAAGTGCATCATCGTTGTTC** |
| ***Mtor*** | **AGAAGGGTCTCCAAGGACGACT** | **GCAGGACACAAAGGCAGCATTG** |
| ***Nes*** | **AGGAGAAGCAGGGTCTACAGAG** | **AGTTCTCAGCCTCCAGCAGAGT** |
| ***Dlg4*** | **TCAGACGGTCACGATCATCGCT** | **GTTGCTTCGCAGAGATGCAGTC** |
| ***Sqstm1*** | **GCTCTTCGGAAGTCAGCAAACC** | **GCAGTTTCCCGACTCCATCTGT** |
| ***Lamp1*** | **CCAGGCTTTCAAGGTGGACAGT** | **GGTAGGCAATGAGGACGATGAG** |
| ***C1qa*** | **GTGGCTGAAGATGTCTGCCGAG** | **TTAAAACCTCGGATACCAGTCCG** |
| ***Syn1*** | **TATGCCACTGCTGAGCCCTTCA** | **ATGGCAATCTGCTCAAGCATAGC** |
| ***Nos2*** | **GAGACAGGGAAGTCTGAAGCAC** | **CCAGCAGTAGTTGCTCCTCTTC** |
| ***C3*** | **CGCAACGAACAGGTGGAGATCA** | **CTGGAAGTAGCGATTCTTGGCG** |
| ***Fpr2*** | **GCCTTTTGGCTGGTTCCTGTGT** | **CAAATGCAGCGGTCCAAGGCAA** |
| ***Actb*** | **CATTGCTGACAGGATGCAGAAGG** | **TGCTGGAAGGTGGACAGTGAGG** |
